# Post-Therapeutic Relapse of Psoriasis after CD11a Blockade Is Associated with T Cells and Inflammatory Myeloid DCs

**DOI:** 10.1371/journal.pone.0030308

**Published:** 2012-02-10

**Authors:** Leanne M. Johnson-Huang, Cara A. Pensabene, Kejal R. Shah, Katherine C. Pierson, Toyoko Kikuchi, Tim Lentini, Patricia Gilleaudeau, Mary Sullivan-Whalen, Inna Cueto, Artemis Khatcherian, Luke A. Hyder, Mayte Suárez-Fariñas, James G. Krueger, Michelle A. Lowes

**Affiliations:** 1 Laboratory for Investigative Dermatology, The Rockefeller University, New York, New York, United States of America; 2 Center for Clinical and Translational Science, The Rockefeller University, New York, New York, United States of America; Blood Systems Research Institute, United States of America

## Abstract

To understand the development of new psoriasis lesions, we studied a group of moderate-to-severe psoriasis patients who experienced a relapse after ceasing efalizumab (anti-CD11a, Raptiva, Genentech). There were increased CD3^+^ T cells, neutrophils, CD11c^+^ and CD83^+^ myeloid dendritic cells (DCs), but no increase in CD1c^+^ resident myeloid DCs. In relapsed lesions, there were many CD11c^+^CD1c^−^, inflammatory myeloid DCs identified by TNFSF10/TRAIL, TNF, and iNOS. CD11c^+^ cells in relapsed lesions co-expressed CD14 and CD16 *in situ*. Efalizumab induced an improvement in many psoriasis genes, and during relapse, the majority of these genes reversed back to a lesional state. Gene Set Enrichment Analysis (GSEA) of the transcriptome of relapsed tissue showed that many of the gene sets known to be present in psoriasis were also highly enriched in relapse. Hence, on ceasing efalizumab, T cells and myeloid cells rapidly enter the skin to cause classic psoriasis.

**Trial registration:**

Clinicaltrials.gov NCT00115076

## Introduction

Efalizumab (anti-CD11a, Raptiva, Genentech) is a fully humanized monoclonal antibody to CD11a, the α-chain of lymphocyte function-associated antigen (LFA-1), which is present on many leukocytes. Although LFA-1 interacts with intracellular adhesion molecules (ICAMS) for leukocyte migration into tissues, antigen-presentation to T cells, and interaction with keratinocytes, the most important therapeutic mechanism of action of efalizumab appears to be via blockade of lymphocyte trafficking [1]. In addition, patients on efalizumab may experience an unusual form of T cell hyporesponsiveness [2], and fail to make effective antibodies to a neoantigen [3].

An investigator-initiated study was conducted to further evaluate the mechanism of action of efalizumab, in 30 patients with moderate-to-severe psoriasis. Patients were given 12 weeks treatment with 1 mg/kg/week subcutaneous efalizumab, followed by a 12-week period of observation. However, 8 of the first 20 patients suffered from a rapid return of their psoriasis in the post-treatment period. The design of the study was subsequently changed to treat the remaining 10 patients with efalizumab for the full 24 weeks of the study. Patients were then transitioned to an alternative treatment at the end of the study, predominantly cyclosporine [4].

The National Psoriasis Foundation Medical Advisory Board has proposed standard definitions of worsening of disease when psoriasis treatment is discontinued, including relapse and rebound [5], [6]. *Relapse* is defined as loss of 50% of Psoriasis Activity and Severity Index (PASI) improvement from baseline in patients who achieve a clinically meaningful response. Relapse in the 12 weeks of follow up after discontinuation of efalizumab therapy was observed in 86% of patients who had achieved a PASI-75. The relapse was often gradual, but some patients experienced a rapid return of disease. *Rebound* is said to occur if the PASI is 125% or greater than baseline, or if new generalized pustular, erythrodermic or more inflammatory psoriasis occurrs within 3 months of stopping therapy. In a pooled analysis of 1316 patients in clinical studies with efalizumab, 188 (14%) experienced a rebound during the 12 week follow-up period [5], [6].

When this study began in 2002, efalizumab was not FDA approved for psoriasis. However, it was subsequently approved in 2003 for the treatment of moderate-to-severe psoriasis vulgaris. Efalizumab was voluntarily withdrawn by Genentech in April 2009 due to reports of several cases of progressive multifocal leukoencephalopathy, a rare and usually fatal infection caused by the reactivation of polyomavirus JC in the central nervous system of immunocompromised individuals [7], [8]. Thus, although this is not an agent in use at the present time, precious clinical material was available to study LFA-1 integrin blockade at a cellular and molecular level.

Eight patients experienced a clinical relapse out of the first 20 patients. Our study demonstrated a marked increase in CD11c^+^ inflammatory dendritic cells (DCs) and T cells, but not macrophages or resident DCs, at the time of disease relapse compared to the end of the treatment period. The present study gives us insights into the mechanism of action of efalizumab, which may be used in the future as an orphan drug, as well as the potential effects of integrin blockade with other agents. Furthermore, this study helps to elucidate the mechanism of new lesion development in psoriasis.

## Methods

The protocol for this trial and supporting CONSORT checklist are available as supporting information; see Checklist S1 and Protocol S1.

### Ethics statement

**Figure 1:**
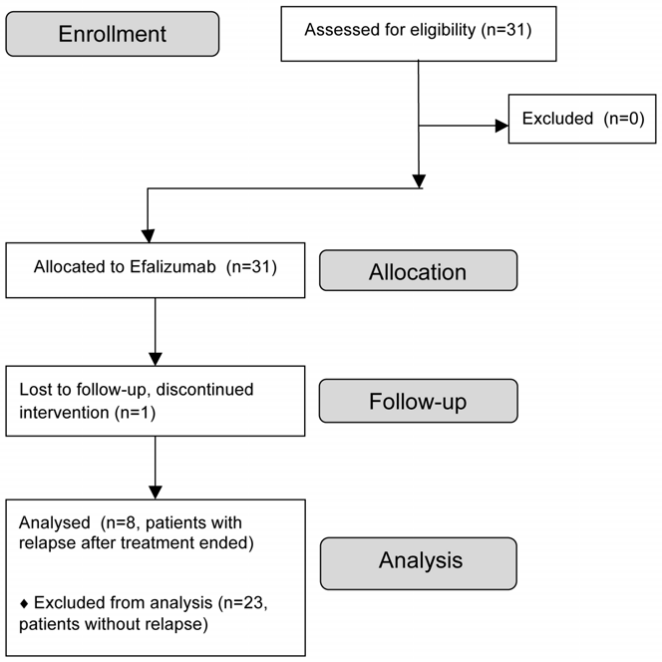
Enrollment flowchart.

Thirty-one adult patients with moderate-to-severe psoriasis vulgaris were enrolled in this Rockefeller University Institutional Review Board-approved study (Figure 1), clinicaltrials.gov NCT00115076 between August 2003 and June 2007. All patients gave written informed consent. Research was conducted in accordance with the Declaration of Helsinki principles.

### Study design

Patients were treated with weekly subcutaneous injections of 1 mg/kg of efalizumab for 12 weeks, and then the first 20 patients were observed weekly for an additional 12 weeks, and the last 10 patients were treated for 24 weeks. Skin biopsies were obtained at baseline from an uninvolved area (non-lesional, NL) and an index psoriatic plaque lesion (LS). Biopsies were also taken from lesions at weeks 2, 6, and 12 of efalizumab treatment. An additional punch biopsy was obtained from patients whose disease worsened after treatment was finished (n = 8).

### Immunohistochemistry

Immunohistochemistry was performed for leukocytes in patients who experienced a relapse (n = 8). Frozen tissue sections were stained with haematoxylin (Thermo Fisher Scientific) and eosin (Shandon) or with murine anti-human monoclonal antibodies as listed in Table S1. Biotin-labelled horse anti-mouse antibodies (Vector Laboratories) were used to amplify the primary signal with an avidin-biotin complex and developed with chromogen-3-amino-9-ethylcarbazole (Sigma Aldrich). Positively stained cells per millimeter were manually counted using image analysis software (Image J, version 1.38×, National Institutes of Health), and reported per mm linear length of the epidermis [9].

### Immunofluorescence

Immunofluorescence was conducted in relapse lesions (n = 3–5) using antibodies and fluorochromes as outlined in Table S1, and as described previously [9]. Images in each figure are presented both as single color stains (green and red) located above the merged image, so that one can appreciate the localization of two markers on similar or different cells. Cells that co-express the two markers in a similar location are yellow in color. A white line denotes the dermo-epidermal junction. Dermal collagen fibers gave green autofluorescence, and antibodies conjugated with a fluorochrome often gave background epidermal fluorescence.

### Microarray

RNA was extracted from full-thickness skin biopsies (NL, LS, week 12 and relapse) of 4 relapsing patients that were the best histological responders (first 4 patients in Table 1). Patients were classified as responders based on normalization of epidermal thickness, keratin 16 (K16) protein expression, restoration of a granular layer, and orthokeratosis in week 12 biopsies [10]. RNA was extracted using the RNeasy Mini Kit (Qiagen, Valencia, CA). For each HGU 133 2.0 Affymetrix gene chip, 2 µg total RNA was reverse transcribed, amplified, and labeled [11]. Experiments were conducted in compliance with Minimum Information About a Microarray Experiment guidelines. Tissue microarray data have been deposited in NCBI's Gene Expression Omnibus and are accessible at GEO Series accession number GSE30768.

**Table 1:** Clinical data and PASI score on the study patients.

Gender	PASIWk 0	PASIWk 12	% Wk 12 to W0^a^	PASI @recurrence	% loss of improvement	Relapse^b^	% change at recurrence	Rebound^c^
F^d^	60	2.7	−96%	46.2	76%	Y	77%	N
M^d^	12.2	3.4	−72%	16.6	150%	Y	136%	Y
F^d^	30.8	9.2	−70%	35.8	123%	Y	116%	N
F^d^	35.2	8.9	−75%	42.9	129%	Y	122%	N
M	24	3.8	−84%	16.5	63%	Y	69%	N
M	35.2	11.4	−68%	36.8	107%	Y	105%	N
M	49.8	8.9	−82%	31.8	56%	Y	64%	N
M	46.2	34.8	−25%	41.2	56%	Y	89%	N

a % change week 12 compared to week 0

b all patients experienced a relapse between weeks 14–22, defined as loss of 50% of PASI improvement from baseline [5], [6].

c only 1 patient experienced a rebound, said to occur if the PASI is 125% or greater than baseline, or if new generalized pustular, erythrodermic or more inflammatory psoriasis occurs within 3 months of stopping therapy [5], [6].

d Genomic characterization on first 4 patients in this table.

The analysis was conducted using R software (http://www.R-project.org) with bioconductor packages (http://www.bioconductor.org). CEL files were scrutinized for spatial artifacts by using the *Harshlight* package [12]. Classic microarray quality control report was obtained by using the *affyQCReport* package. Expression values were obtained by using GCRMA algorithm. Probe-sets with standard deviation (SD) greater than 0.1 and expression values greater than 3 in at least 2 samples were kept for further analysis (13405 probe-sets). Expression values were modeled using mixed effect models with Time as fixed effect and Patient as a random factor. Model estimation and hypothesis testing were conducted in the framework of *limma.* Comparisons of interest were assessed using moderated-t test, and resulting p-values were adjusted for multiples hypothesis using Benjamini-Hochberg approach. Probe-sets with fold change (FCH) larger that 2 and false discovery rate (FDR) smaller than 0.05 were selected as efalizumab modulated genes.

RT-PCR was conducted for keratin 16 (K16) as previously described [13].

### Flow cytometry

PBMCs were stained with CD11a-FITC (clone 25.3.1, binds to an alternative epitope than the efalizumab-antibody binding site, Immunotech) and CD14-APC (BD) in a standard manner.

### Statistics

Mean PASI, absolute lymphocyte count, epidermal thickness, and cell count data were analyzed using a Freidman test (paired 1 way ANOVA). Median fluorescence intensity for CD11a expression by flow cytometry was analyzed using a paired Wilcoxon signed rank test. Significance was accepted as p<0.05.

## Results

### Characteristics of patients who experienced a relapse

**Figure 2:**
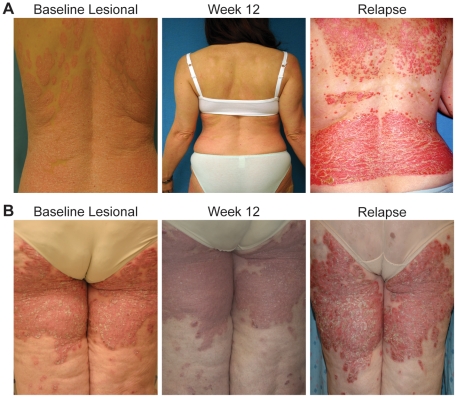
Clinical photographs of two representative patients with disease relapse upon cessation of efalizumab treatment. (**A**) Baseline lesions (left), response to efalizumab (center) and a marked erythematous psoriatic reaction 5 weeks after ceasing treatment (right). (**B**) Baseline photographs showing upper leg psoriatic plaques (left) which responded to efalizumab at week 12 (center) and a relapse in the same location (right). Note the inflammatory nature of the lesions during relapse.

Eight patients experienced a relapse at weeks 14–22, and were included in this study. Table 1 summarizes details of these patients. Three patients were female, and five were male; the majority were Caucasian (7/8), and the other patient was Hispanic. Figure 2A and 2B demonstrate two patients with disease relapse, showing the inflammatory nature of the relapse.

**Figure 3:**
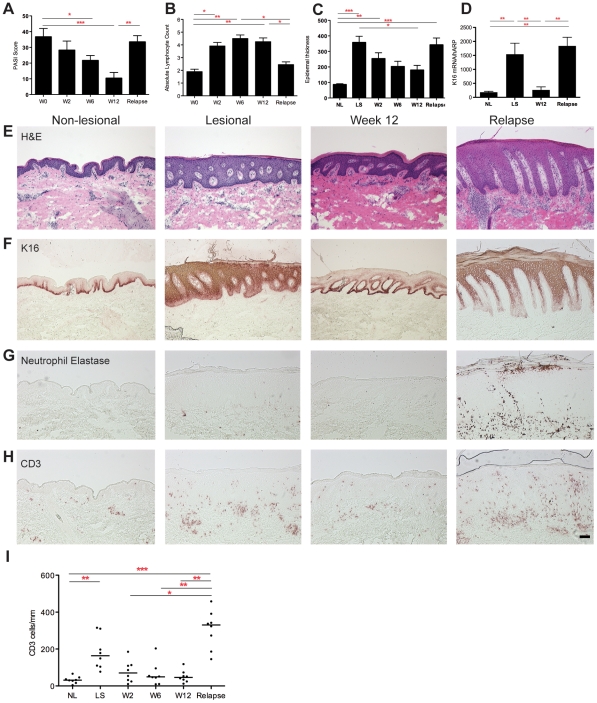
Clinical and histological response to efalizumab and during relapse. (**A**) Mean PASI scores, (**B**) circulating absolute lymphocyte count, (**C**) epidermal thickness (µm), (all n = 8) and (**D**) normalized keratin 16 (K16) mRNA expression (n = 4), throughout the trial and at time of relapse. Non-lesional (NL) and lesional skin (LS), error bars represent the standard error of the mean. ^*^ p<0.05; ^**^ p<0.01; ^***^ p<0.001. Representative (**E**) haematoxylin and eosin (H&E), (**F**) K16 protein, (**G**) neutrophil elastase, (**H**) CD3^+^ T cell immunohistochemistry, and (**I**) CD3^+^ T cell counts, showing the psoriasiform nature of the relapse lesion, with characteristic K16 staining, neutrophils and CD3^+^ T cells. Bar is 100 µm.

Mean PASI at baseline was 36.7, which was reduced to mean 21.7 by week 6 (p<0.05), and 10.4 at week 12 (p<0.001), representing a mean decrease of 72% (Figure 3A). Four patients met or exceeded *PASI 75*, and 7 exceeded *PASI 50*. At relapse, the mean PASI was 33.5, which was significantly increased compared to week 12 (p<0.01). All patients experiencing a recurrence of disease after ceasing treatment met the criteria for *relapse*, which is defined as a loss of 50% of PASI improvement over baseline. One patient also met the criteria for *rebound*, defined as PASI 125% or greater of baseline within 3 months of stopping therapy. Patients who do not respond to therapy are actually more likely to experience rebound [6], but in our cohort, the patient who experienced rebound had a *PASI 72* score. Those patients who had disease relapse were treated with cyclosporine and/or restarted efalizumab therapy.

### Minimal residual effect of efalizumab at the time of relapse

During treatment with efalizumab the absolute lymphocyte count was measured by calculating total white blood cell count times the percentage of lymphocytes, based on complete blood count data [1]. At the end of treatment, there was an approximately two-fold increase in the mean absolute lymphocyte count compared to baseline (Figure 3B), indicative of an effect of circulating efalizumab [1]. At the time of relapse there was a significant reduction in circulating lymphocytes compared to week 12 of treatment (p<0.05), as efalizumab was cleared from the circulation during this observation period and the lymphocytosis was reversed. CD11a expression on monocytes (in the six patients that could be measured) was reduced from baseline to week 12 from a mean of 105 to 66 (p = 0.06) and was significantly re-expressed at relapse to 121 (p = 0.03). The normalization of the lymphocyte count and the re-expression of CD11a at the time of relapse indicate that efalizumab was no longer exerting an effect, and thus the newly available CD11a now allows leukocytes to move into the skin during relapse.

### Histological responses

Epidermal thickness was measured during therapy and at the time of relapse (Figure 3C). There was a significant increase in the epidermal thickness in LS compared to NL skin (p<0.001), improvement with treatment (p<0.05), and an increase at relapse compared to week 12, although non-significant. K16 mRNA expression was similarly increased in LS skin, decreased with treatment at week 12, and increased again with relapse (all p<0.01) (Figure 3D). Representative histological effects of efalizumab (Figure 3E) and K16 staining (Figure 3F) demonstrate normalization of psoriatic inflammation, and K16 expression by week 12, and the histological features of relapse, which are clinically consistent with psoriasis.

Leukocytic infiltration during treatment with efalizumab and at the time of relapse was next evaluated (Figure 3G–I). Neutrophil elastase was increased in relapse (Figure 3G), indicating the presence of abundant neutrophils as the lesions returned, similar to florid inflammatory or pustular psoriasis. CD3^+^ T cells were increased in relapse compared to the end of treatment (p<0.01, representative staining in Figure 3H, cell counts in Figure 3I). There was a negative correlation between lymphocytes in the blood and cutaneous CD3^+^ T cells (Spearman correlation co-efficient r = −0.5, p = 0.0012) (Figure S2A). CD163^+^ macrophages were not increased in relapse compared to the end of treatment or lesional skin (data not shown).

### Characterization of inflammatory myeloid DCs in psoriasis lesions

**Figure 4:**
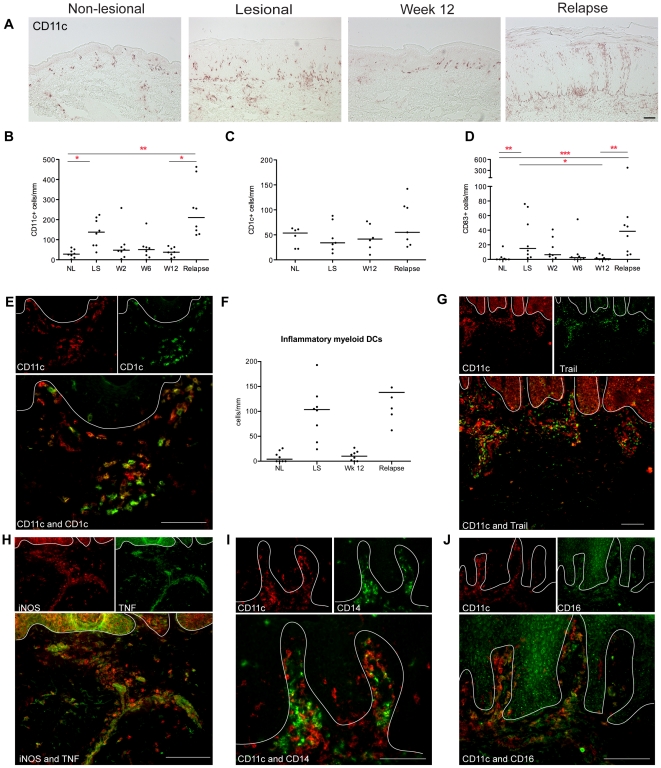
Increased inflammatory myeloid DCs in relapsed lesions. (**A**) Representative immunohistochemistry and (**B**) counts of CD11c^+^ cells per mm in non-lesional skin (NL), lesional skin (LS), and in the index lesional plaque at weeks 2, 6 and 12 and time of disease relapse. (**C**) The numbers of CD1c^+^ cells did not change with treatment or relapse. (**D**) CD83^+^ mature DCs followed the same pattern as CD11c^+^ DCs. (**E**) There were many inflammatory myeloid DCs in the relapsed lesions, shown by immunofluorescence as CD11c^+^ cells (red) that were not co-expressing CD1c (and thus were not yellow), and (**F**) quantified as CD11c^+^ minus CD1c^+^ cells. (**G**) Inflammatory CD11c^+^ DCs were co-expressing TNFSF10/TRAIL. (**H**) TNF- and iNOS-producing DCs (TIP-DCs) were found in relapsed lesions. CD11c^+^ cells (red) co-expressing (**I**) CD14 and (**J**) CD16 (both green). Cells that co-express the two markers in a similar location are yellow in color. A white line denotes the dermo-epidermal junction. Bar is 100 µm.

CD11c is the most useful and general marker of cutaneous myeloid DCs [14], and representative staining for these cells is shown in Figure 4A with cell counts in Figure 4B. At the time of relapse, CD11c^+^ myeloid DCs were increased in lesions compared to the end of treatment (p<0.05), and also above NL levels (p<0.01) (Figure 4B). Other useful markers for myeloid DCs are blood dendritic cell antigen (BDCA)-1/CD1c, which identifies a population of resident DCs, and the mature DC marker CD83. Consistent with previous anti-psoriasis treatments [10], [15], BDCA-1/CD1c did not change with disease state, efalizumab treatment or relapse (Figure 4C). However, CD83^+^ cells were increased in LS skin, decreased with treatment, and increased with relapse (Figure 4D).

We have defined “inflammatory” myeloid DCs as CD11c^+^ CD1c^−^ cells [16], [17]. Two-color immunofluorescence identified many CD11c^+^ cells (red) that were not CD1c^+^ (green) (Figure 4E). Cells expressing both colors are seen as yellow, which are the resident population of dermal myeloid DCs. The frequency of these inflammatory myeloid DCs can be calculated by subtracting CD1c cell counts from CD11c [15], and there was an increase in CD11c^+^CD1c^−^ cells at relapse (p<0.06) (Figure 4F).

We have recently identified that TRAIL/TNFSF10 is a marker of these inflammatory myeloid DCs [18], and TRAIL co-localizes with many CD11c^+^ cells (Figure 4G). TNF and iNOS-producing DCs (TIP-DCs) [16] are considered to be a subset of inflammatory myeloid DCs in psoriasis [17], and there were abundant TNF and iNOS producing cells (yellow cells) in lesional tissue at relapse (Figure 4H). Thus, during a relapse, cells were present that have been previously identified as integral cellular components of a psoriasis lesion.

As murine studies have shown that a circulating monocyte population may be precursors for TIP-DCs [19], two-color immunofluorescence was also performed for CD11c versus CD14 and CD16. CD14^+^ cells were increased in relapsed lesions (p<0.05, Figure S2B). We have previously shown that both these markers are present on some CD11c^+^ cells [9], and this was confirmed in relapsed lesions (Figure 4I, J).

### Genomic characterization of relapse

**Figure 5:**
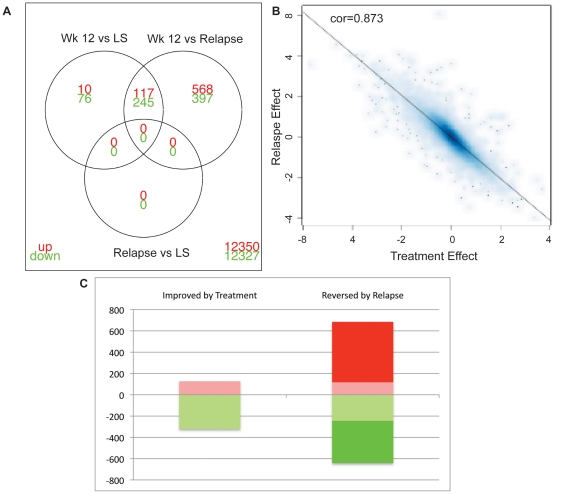
Genomic characterization of relapsed psoriasis lesions. (**A**) Venn diagram showing DEGs for different comparisons, week 12 versus LS; week 12 versus relapse; relapse versus LS. There were no unique DEGs in relapse compared to LS skin. (**B**) Scatter plot showing an excellent correlation between the “treatment effect” and “relapse effect”. (**C**) Number of genes improved by treatment and reversed by relapse. Red blocks were increased DEGs, green blocks were decreased DEGs.

In order to further characterize the skin during relapse, samples from four patients who responded to treatment and experienced a relapse were hybridized to Affymetrix HGU 133 2.0 expression chips (first four patients in Table 1). We explored the difference between any of the time points (NL, LS, week 12 and relapse), at FCH≥2 and FDR<0.05, presented in Table S2. Unsupervised clustering of the expression data for these DEGs (compared to relapse) showed the first level of clustering of the NL and week 12 biopsies together, and second cluster with the LS and relapse biopsies (Figure S1). There were no differentially expressed genes between LS and relapsed skin (Figure 5A). The treatment effect (Week 12 versus LS) and the Relapse effect (Relapse versus Week12) were quantified and showed an excellent correlation (Figure 5B). In terms of magnitude, change induced by treatment was highly correlated with the relapse effect (r = −0.87,p<10^16^, Adjusted R^2^: 0.76), with a slightly larger effect in the relapse (64% of the genes had larger effect in Relapse than Treatment). Almost all genes that were improved by treatment were returned to LS levels in the relapsed skin: 92% of the genes up-regulated by efalizumab and 76% of the down-regulated genes were reversed after relapse, but the relapse effect encompassed a larger number of genes (Figure 5C). This indicates that the relapse at the genomic levels is a return to psoriasis, but even “worse”.

To better understand the transcriptome generated by these studies, we used Gene Set Enrichment Analysis (GSEA) to identify gene sets that were related to the psoriasis relapse phenotype (Relapse versus Week 12) (Table 2), as we have done previously for the psoriasis phenotype (LS versus NL) [20]. The closer the enrichment score (ES) to 1, the closer the ranking of the genes in the gene set to those in the list being queried. When upregulated (UP) and down-regulated (DOWN) gene sets are both enriched, a connectivity score (CS) can be calculated, and again, the closer to 1, the higher the ranking.

**Table 2:** GSEA analysis of published gene sets.

PATHWAY/GENE SET	SIZE^a^	ES^b^	NES^c^	p-val	FDR	CS^d^	REF^e^
**LS vs NL:**							
PSORIASIS_SF_UP	551	0.88	3.89	0.000	0.000	0.86	[20]
PSORIASIS_SF_DOWN	680	−0.83	−4.29	0.000	0.000		
**Relapse vs Week 12:**							
PSORIASIS_SF_UP	569	0.85	4.26	0.000	0.000	0.84	[20]
PSORIASIS_SF_DOWN	694	−0.82	−4.25	0.000	0.000		
IL22 KC_UP	11	0.86	2.16	0.000	0.000	0.84	[21]
IL22 KC_DOWN	10	−0.82	−2.05	0.000	0.000		
IFNG KC_UP	907	0.42	2.19	0.000	0.000	0.34	[21]
IFNG KC_DOWN	332	−0.25	−1.23	0.050	0.128		
IL17&TNF KC UP	31	0.87	2.82	0.000	0.000		[22]
IL1 KC_UP	48	0.75	2.68	0.000	0.000		[23]
TERMINAL DIFFERENTIATION	33	0.58	1.86	0.000	0.001		[24]
PS_INFLAMM DCS_UP	131	0.33	1.41	0.008	0.041		[18]

a Size, number of genes in gene set.

b ES, enrichment score.

c NES, normalized enrichment score.

d CS, connectivity score.

e REF, reference for gene set.

GSEA analysis of the LS versus NL skin genes showed that the patients in our study had typical or classic psoriasis at the time of enrollment, as there was a high connectivity score (0.86) for genes in this list compared to a published list of DEGs by our group [20]. In the relapse phenotype, there was a high connectivity score (CS) for the psoriasis transcriptome (“PSORIASIS_SF”) [20] and many of the gene sets that have been shown to be enriched in psoriasis [20]. This included the gene sets of keratinocytes treated with IL-22 (“IL22 KC”) and IFNγ (IFNG KC”) [21]; up-regulated genes in keratinocytes treated with IL-17 plus TNF (“IL17&TNF KC_UP”) [22], and IL-1 (IL1 KC_UP”) [23]; and the terminal differentiation gene set [24] was also enriched in relapse. Furthermore, the gene set for inflammatory myeloid DCs [18] was also enriched in the relapse transcriptome. Thus, relapse is akin to a severe psoriasis clinical phenotype.

## Discussion

Our study showed that in skin lesions that develop during relapse when efalizumab is ceased, there were abundant T cells and DCs. These are leukocytes commonly found in psoriasis lesions [25]. The genomic transcriptome of relapse is also very similar to lesional psoriasis skin [20]. These data suggest that the lesions of relapse are typical psoriasis, in contrast to lesions that develop while *on* therapy, which have reduced cutaneous T cells [26]. Furthermore, this investigation supports current knowledge of requisite cells for the development of a psoriasis lesion, namely T cells and antigen-presenting cells, with all the products that they make.

This study also supports our developing concept that there is a resident myeloid DC population in normal skin, marked by BDCA-1/CD1c, which does not change in number during inflammation [10], [15]. CD11c has been used as a marker of interstitial myeloid DCs [27]. The numbers of CD11c^+^ cells in psoriasis are many fold greater than CD1c^+^ cells, as CD11c identifies both the resident myeloid DCs and a population of “inflammatory” myeloid DCs. In contrast to resident CD1c^+^ DCs, CD11c^+^ cells are increased in psoriasis, reduced with treatment, and increased during relapse. We have previously shown that inflammatory DCs are important pathogenic cells in psoriasis, as they produce TNF, iNOS, IL-20, and IL-23, and are TRAIL^+^ [10], [11], [16], [17], [18]. Nitric oxide produced by iNOS activity may explain the marked erythema of these relapse lesions. These data, and the observation that CD163^+^ cells were not increased, suggest a more critical role for inflammatory DCs than macrophages in the relapsed psoriasis lesion.

CD11a is a component of the heterodimeric β_2_-integrin LFA-1, which is essential in transmigration of leukocytes into tissues during inflammation and homeostasis. The monoclonal antibody efalizumab binds CD11a, blocks the function of the LFA-1 molecule binding to ICAMs, and prevents CD11a^+^ leukocytes from leaving the circulation, effectively trapping them there. This explains the peripheral leukocytosis that occurs in all patients taking adequate doses of efalizumab [1], which we also saw in our patients. The most prominent mononuclear cells comprising the leukocytosis are CD8^+^ T cells, and there was also an increase in circulating IFN-γ^+^ T cells during treatment [1]. IL-17-producing T cells were not studied at that time as their role in autoimmune diseases was just being appreciated. Thus, when the drug is stopped suddenly, there is a reservoir of CD11a^+^ CD8^+^ T cells in the circulation ready to pour into the skin leading to rapid onset of severe psoriasis.

Our biopsies were taken when relapsed patients presented with florid lesions, so the above model does not fully explain the kinetics of how new lesions of psoriasis develop. It is possible that CD8^+^ IFN-γ-producing T cells enter the skin first, as they are “backed-up” in the circulation, setting off a psoriatic inflammatory cascade via DC activation, as proposed by Kryczek *et al* [28]. These CD8^+^ T cells induce chemokines for circulating DC-precursors, such as monocytes, to enter the skin and become inflammatory myeloid DCs. There is then an amplifying cycle of DCs and skin homing memory T cells migrating into the skin to drive lesion development.

The concept of monocytes as precursor cells for inflammatory DCs has been well studied in mice [29], with a relevant example being Gr-1^hi^ CCR2^+^ monocytes giving rise to TIP-DCs in the spleens of mice infected with *Listeria monocytogenes* [19]. An increase in CD14^+^ cells, and co-localization of CD14 and CD16 with CD11c^+^ myeloid DCs *in situ* in our study, suggests that monocytes may be precursor cells giving rise to the inflammatory DCs. However, in contrast to lymphocytes, there was no direct relationship between circulating monocytes, which remained constant, and skin CD14^+^ cells, whose pattern mirrored T cells. At present, it is unclear both what circulating CD14^+^ monocytes become once they enter the tissues, and also exactly which myeloid subset is the precursor of tissue inflammatory myeloid DCs. As the monocytes quickly migrate into the skin, they may retain CD14 expression, which may explain why inflammatory DCs express monocyte markers CD14 and CD16, but normal skin myeloid DCs do not [9]. While there is a minor population of CD14^+^ DCs in normal skin that has been proposed to drive Th2 responses [30], the identity and role of CD14^+^ in psoriasis has not been specifically studied, and CD14^+^ cells may be a heterogeneous population of either myeloid DCs or macrophages. Subsets of circulating CD14^+^ cells may give rise to any or all of these populations of tissue-resident myeloid cells.

The mechanism by which myeloid DC precursors enter the skin is also not entirely known. CD14^+^ monocytes were not increased in the circulation while patients were on efalizumab [1], but there was a trend of reduced expression of monocyte CD11a during treatment, with significant increase in surface CD11a by the time of relapse. Hence there is a pool of circulating monocytes with available surface CD11a that can access the tissues at the time of relapse. Secondly, there was increased genomic expression of many myeloid cell chemokines at the time of relapse, namely CCL2, CCL20, CXCL1, CXCL2, IL-8/CXCL8 (Table S1, Figure S2C). CCL2 is a known monocyte chemokine, and indeed the development of inflammatory DCs in mice is dependant on CCR2, the CCL2 receptor [31]. There is abundant CCL20 protein staining in psoriasis, which is chemotactic for CCR6^+^ immature DCs and Th17 cells [32], [33]. Monocytes from patients on efalizumab were shown to be deficient in their chemotaxis to IL-8, but they responded to chemokines normally in the absence of efalizumab [34]. These data suggest that monocyte chemokines increase during the period after ceasing efalizumab, and perhaps reach a threshold to set up a chemotactic gradient for inflammatory myeloid DC precursors and memory Th17 cells to enter the skin.

Increased numbers of immune cells in relapse lesions suggests that releasing the CD11a blockade upon cessation of efalizumab treatment creates a rapid influx of these leukocytes into the skin, resulting in the development of new psoriatic lesions in some patients. It is as if efalizumab applies a brake to inflammatory leukocytes, retaining them in the circulation, and when efalizumab is ceased, the brake is released. As these cells enter the skin abruptly over a few weeks, they may cause an inflammatory psoriasis that is difficult to treat, usually requiring an additional systemic agent. In our study, 40% of patients experienced severe relapse. Our findings suggest that the major mechanism of action of efalizumab is to “hold” pathogenic leukocytes in the circulation.

Relapsed psoriasis lesions that develop in the setting of efalizumab withdrawal appear to be true psoriasis lesions, with all the complement of leukocytes and gene expression found in stable plaque psoriasis. At this stage, it is not clear which cell type is leading the way, but both antigen-presenting cells, such as the CD11c^+^ inflammatory myeloid DCs and T cells, are required. When efalizumab treatment is withdrawn, the release of the CD11a blockade causes a rapid influx of immune cells into the skin, resulting in the development of new psoriatic lesions in some patients. In the clinical setting of ceasing efalizumab treatment, we have termed this mechanism of cellular infiltration “release of brake”.

## Supporting Information

Figure S1 **Heatmap of differentially expressed genes (DEGs).**
(TIF)

Figure S2 **Circulating versus lesional T cells, CD14+ lesional cells, and expression of chemokines.**
(JPG)

Table S1 Antibodies for Immunohistochemistry and **I**mmunofluorescence.
(DOC)

Table S2 Differentially expressed genes (DEGs) for patients that experienced a relapse after ceasing efalizumab treatment, between any of the time points (NL, LS, week 12 and relapse), at FCH≥2 and FDR<0.05.
(XLS)

Checklist S1 CONSORT Checklist.
(DOC)

Protocol S1 Clinical trial protocol.
(DOC)
