# Supplementary figures and images for: Post-Therapeutic Relapse of Psoriasis after CD11a Blockade Is Associated with T Cells and Inflammatory Myeloid DCs
Source: PLoS One. 2012 Feb 10;7(2):e30308. doi: 10.1371/journal.pone.0030308 (PMC3277585; doi:10.1371/journal.pone.0030308)

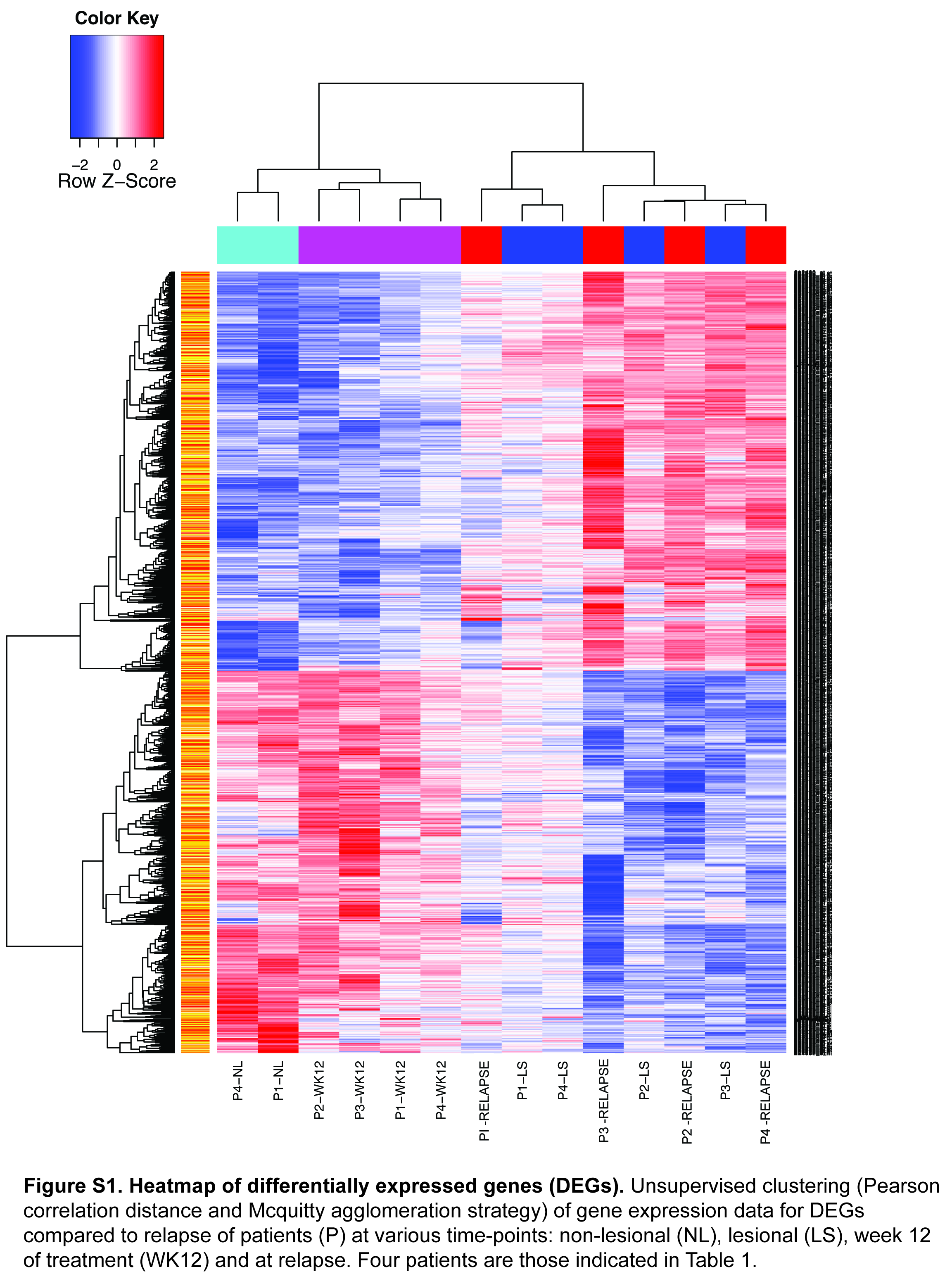

Supplement: Figure S1 — Heatmap of differentially expressed genes (DEGs). (TIF) [file pone.0030308.s001.tif]

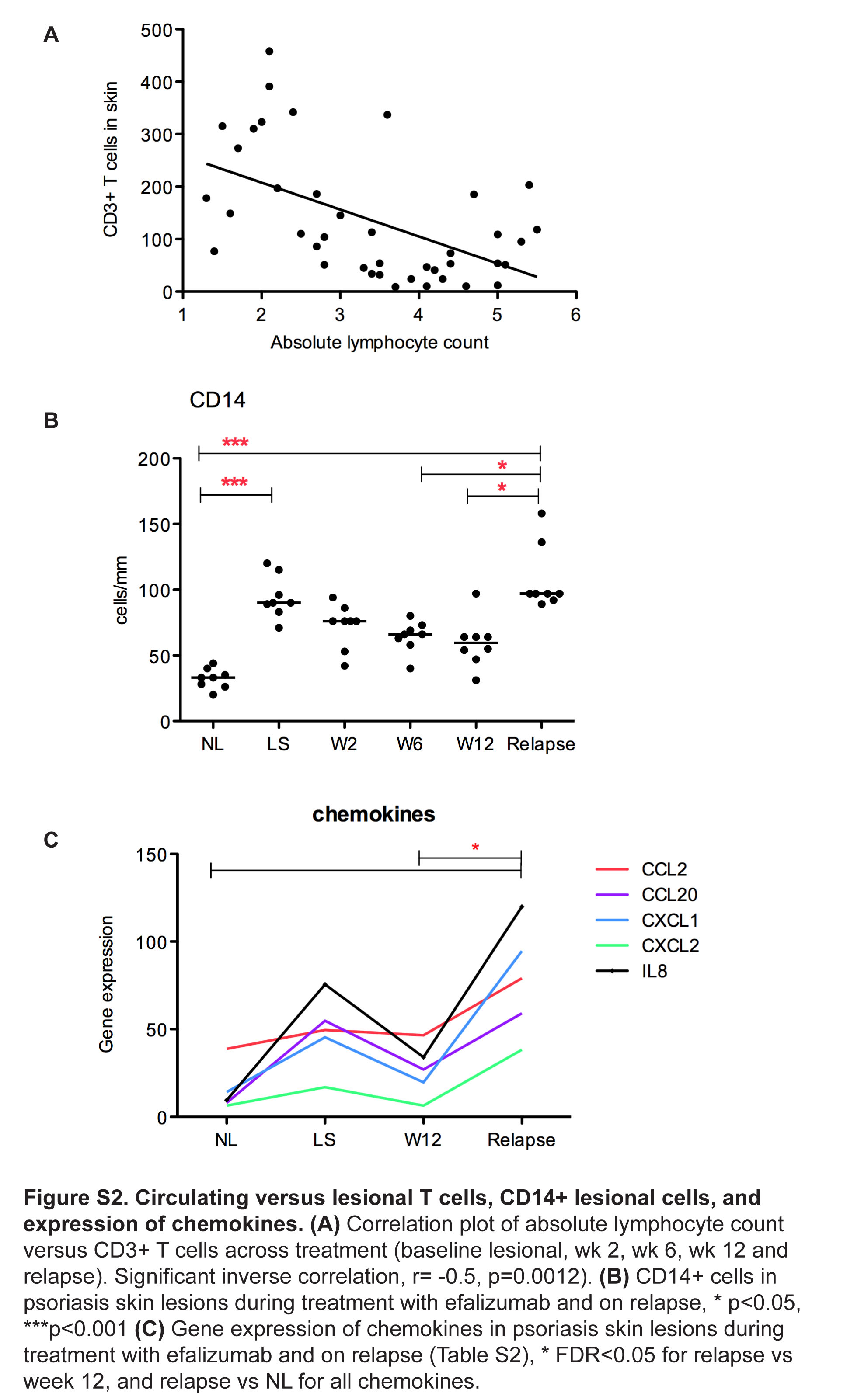

Supplement: Figure S2 — Circulating versus lesional T cells, CD14+ lesional cells, and expression of chemokines. (JPG) [file pone.0030308.s002.jpg]
