# Supplementary material for: Post-Therapeutic Relapse of Psoriasis after CD11a Blockade Is Associated with T Cells and Inflammatory Myeloid DCs
Source: PLoS One. 2012 Feb 10;7(2):e30308. doi: 10.1371/journal.pone.0030308 (PMC3277585; doi:10.1371/journal.pone.0030308)
Supplement: Table S1 — Antibodies for Immunohistochemistry and Immunofluorescence. (DOC) [file pone.0030308.s003.doc]

Table S1. Antibodies for Immunohistochemistry and **I**mmunofluorescence

| Antigen | **Manufacturer** | **Clone**^a^ | **Iso** | **Dil** | **Amplification/ detection**^b^ |
| --- | --- | --- | --- | --- | --- |
| K16 | Sigma-Aldrich | K8.12 | IgG1 | 1:1000 | N/A |
| CD3 | BD Pharmingen | SK7 | IgG1 | 1:100 | N/A |
| CD163 | Acris | 5C6-FAT | IgG1 | 1:100 | Goat anti-mouse IgG1-A568 |
| neutrophil elastase | Dako | NP57 | IgG1 | 1:200 | N/A |
| CD11c | BD Pharmingen | B-ly6 | IgG1 | 1:100 | Goat anti-mouse IgG1-A568 |
| CD83 | Immunotech | HB15a | IgG2b | 1:50 | Goat anti-FITC-A488 |
| INOS | Santa Cruz Biotechnology | Rabbit polyclonal N-20 | IgG | 1:20 | Chicken anti-rabbit IgG-A594 |
| TNF-α (FITC) | BD Biosciences | 6401.1111 | IgG1 | 1:10 | Goat anti-FITC-A488 |
| BDCA-1/CD1c | Miltenyi Biotech | AD5-8E7 | IgG2a | 1:100 | Goat anti-mouse IgG2a-A488 |
| TRAIL/TNFSF10 | R&D Systems | 75402 | IgG1 | 1:50 | goat anti-mouse IgG1-Alexa 488 |

^a^All are murine monoclonals unless stated

^b^All amplification/ detection antibodies are from Invitrogen /Molecular Probes

.
