# Supplementary material for: Post-Therapeutic Relapse of Psoriasis after CD11a Blockade Is Associated with T Cells and Inflammatory Myeloid DCs
Source: PLoS One. 2012 Feb 10;7(2):e30308. doi: 10.1371/journal.pone.0030308 (PMC3277585; doi:10.1371/journal.pone.0030308)
Supplement: Protocol S1 — Clinical trial protocol. (DOC) [file pone.0030308.s006.doc]

# Research Study Submission to the

**Institutional Review Board of The Rockefeller University**

**1. Principal Investigators:** James G. Krueger, MD PhD

**2. Investigators Who Will Conduct the Study:**

Patricia Gilleaudeau, RN, MSN, FNP

Mary Sullivan-Whalen, RN, MSN, FNP

Michelle Lowes, MD, PhD

Toyoko Kikuchi, MA, Flow Cytometry

1. **Title of Study:** A Phase IIIB Study to Evaluate the Mechanism of Action of 1.0 mg/kg Subcutaneously Administered Efalizumab in Adults with Moderate to Severe Plaque Psoriasis who are Candidates for Systemic Therapy
2. **Grant(s) in Which This Study is described**: NIH Grant # 5 R01 A149572, Therapeutic immune mechanisms of anti-CD11a in psoriasis
3. **Type of Study:** a study submitted for renewal
4. **Date of Study:**  ongoing

**7. Other Institutions Involved:** none

**8. Investigational New Drugs Involved:**

None, Now FDA approved

**9. Radioactive Isotopes Involved:** No.

**10. Abstract, Rationale of Study, Research Plan and Procedures:**

- **Abstract**

This is a 24-week study with Efalizumab, a biologic agent injected subcutaneously once weekly. The dose administered is 1 mg/kg of body weight. Patients are seen weekly for the first 12 weeks and they are injected with the medication in our outpatient clinic. Patients are instructed to mixing and self-administering the medication to themselves, and administer the medication to themselves for the second 12 weeks of the study. They are then seen every other week in the outpatient clinic. Skin biopsies are performed before, during, and after the study. Clinical assessments and photography will parallel the skin biopsies. Patients will be evaluated at each visit for any adverse events and safety labs will be taken monthly.

- **Rationale of Study, Research Plan and Procedures**

**A) Overview:**

background

The interaction of T lymphocytes with antigen‑presenting cells (APCs) is one of the initial steps in the activation of an immunologic response to what is perceived by the immune system to be a foreign antigen. Although much attention has been focused on the primary interaction of the T‑cell receptor with the major histocompatability complex (MHC)‑antigen on the APC, several other cell surface components are also involved in, and necessary for, T‑cell activation. These ligand pairs, located on the cell surface of the T-cell and the APC, respectively, include lymphocyte function–associated antigen (LFA)‑1/intercellular adhesion molecule (ICAM)‑1 (also ICAM‑2 and ICAM‑3), CD28/B7, CD2/LFA‑3, CD4/MHC Class II, and CD8/MHC Class I. Interference with the binding of any of these ligand pairs (e.g., with the use of monoclonal antibodies) may decrease or inhibit T‑cell responses (Kuypers and Roos 1989; Dustin and Springer 1991).

LFA‑1 (consisting of CD11a and CD18 subunits) interaction with ICAM is necessary for T‑cell killing, T‑helper and B‑cell responses, natural killing, and antibody‑dependent cytotoxicity. In addition, LFA‑1/ICAM interactions are involved in adherence of leukocytes to endothelial cells, fibroblasts, and epithelial cells, which facilitates migration of leukocytes from the vasculature to the sites of inflammation (Fischer et al. 1986; Springer et al. 1987).

Use of antibodies that interfere with LFA‑1/ICAM interactions could decrease or inhibit the inflammatory process by blocking activation of T-cells and/or the extravasation of leukocytes. Monoclonal antibodies against LFA‑1 or its ligands inhibit T‑cell activation in vitro (Krensky et al. 1983; Dustin and Springer 1988), inhibit T-cell–dependent B‑cell proliferation (Benjamin et al. 1988), target T‑cell lysis (Tanaka et al. 1995), and reduce adhesion of T-cells to the vascular endothelium (Gordon et al. 1995). In mice, anti-CD11a antibodies have been shown to induce tolerance to protein antigens (Nakakura et al. 1993; Cavazzana‑Calvo et al. 1995), delay the onset and reduce the severity of experimental autoimmune encephalomyelitis (He et al. 1994; Gordon et al. 1995), inhibit lupus‑associated autoantibody production, and prolong survival of several types of tissue and organ allografts (Cobbold et al. 1990; van Dijken et al. 1990; Isobe et al. 1992; Talento et al. 1993; Khazaeli et al. 1994; Nishihara et al. 1995; Werther et al. 1996; Bauer 2000; Gottlieb et al. 2000).

Psoriasis is an inflammatory disease characterized by hyperproliferation of keratinocytes and accumulation of activated T-cells in the epidermis and dermis of psoriatic lesions. Patients with psoriasis have successfully responded to treatment with various immunomodulatory or immunosuppressive agents (e.g., cyclosporine and methotrexate). The upregulation of ICAM‑1 on keratinocytes and its interaction with T‑cell LFA‑1 in lesional skin (Nickoloff et al. 1990) indicate that treatment with an antibody such as efalizumab might interfere with the disease process in psoriasis.

Efalizumab is a humanized immunoglobulin (Ig)G1 version of the murine efalizumab monoclonal antibody MHM24, which recognizes human and chimpanzee CD11a. Humanization of MHM24 was accomplished by grafting the murine complementarity determining regions (hypervariable region) into consensus human IgG1/ heavy‑ and light‑chain sequences (Werther et al. 1996). These same consensus human Ig sequences have been successfully used in the humanization of other murine antibodies, including those targeted to HER2 and IgE. Efalizumab blocks T-cell–dependent functions mediated by LFA‑1, including inhibition of the mixed lymphocyte response to heterologous lymphocytes and adhesion of human T-cells to keratinocytes.

Nonclinical EXPERIENCE with efalizumab

Eleven nonclinical studies have been performed to assess the safety, toxicology, pharmacokinetics, pharmacodynamics, and immunogenicity of efalizumab. Efalizumab was generally well tolerated in chimpanzees at doses of up to 40 mg/kg/wk administered for 26 weeks. Although the death of one chimpanzee was attributed to a chronic infection not associated with efalizumab treatment, a potential exacerbation of the infection by efalizumab cannot be ruled out. Administration of efalizumab at 38 mg/kg/wk resulted in a significant reduction in the humoral immune response; however, this effect appears to be reversible upon clearance of the drug.

Re-exposure to a single dose of efalizumab (up to 10 mg/kg) was well tolerated in chimpanzees. Only one chimpanzee in the toxicology studies developed an anti-efalizumab antibody response, but no adverse effects associated with this antibody response were noted in this animal. Cellular expression of CD11a was down‑regulated following efalizumab administration, and re‑expression occurred 7-10 days after efalizumab was eliminated from the blood.

The pharmacokinetics and pharmacodynamics of efalizumab in chimpanzees were similar to those seen in humans. Data from all species tested support that binding to CD11a plays an important role in the absorption, distribution, metabolism, and excretion of efalizumab. Together, these data were consistent with the understanding of the mechanism of action across species, and the doses selected were appropriate for understanding the effect of the proposed clinical doses.

For further information, see the Investigator Brochure.

Clinical Experience with Efalizumab

Fourteen clinical studies have been undertaken to evaluate the safety, pharmacokinetics, pharmacodynamics, and efficacy of efalizumab in subjects with moderate to severe plaque psoriasis, and one study has been conducted in healthy volunteers (see Table 1). Eleven trials have been completed. Three of these trials employed intravenous (IV) administration of efalizumab: a single‑dose, dose‑escalation study (HU9602; Werther et al. 1996; Bauer et al. 1999; Gottlieb et al. 2000); a multidose, dose‑escalation study (HUPS249); and a randomized, double‑blind, placebo‑controlled study (HUPS252). Three studies (HUPS254, HUPS256, and ACD2142g) examined the safety, tolerability, pharmacokinetics, pharmacodynamics, and efficacy of subcutaneously administered efalizumab. Three double‑blind, placebo‑controlled, randomized, multicenter, Phase III studies (ACD2058g, ACD2059g, and ACD2390g) investigated treatment in subjects with moderate to severe plaque psoriasis. A fourth open-label study (ACD2062g) has been completed. One study (ACD2389g) was a single-dose comparability study of Genentech‑ and XOMA-manufactured efalizumab in healthy volunteers. One other double-blind, placebo‑controlled, Phase III study (ACD2600g) and three open‑label studies (ACD2243g, ACD2391g, and ACD2601g) are in progress.

| Table 1  Efalizumab Clinical Studies in Psoriasis Program | | | |
| --- | --- | --- | --- |
| Study (Phase) | Status | Subjects ^a^ | Dose (mg/kg)/ Route/Frequency/Duration |
| HU9602 (Ia) | Completed | 31 | 0.03, 0.1, 0.3, 0.6, 1.0, 2.0, 3.0, or 10.0/ IV/single dose |
| HUPS249 (Ib) | Completed | 39 | 0.1, 0.3, 0.6, or 1.0/IV/every other week or weekly/4–7 weeks |
| HUPS254 (Ic) | Completed | 57 | 0.3, 0.5, 0.7, 1.0, 1.5, or 2.0/SC/weekly/ 1–8 weeks |
| HUPS256 (Id) | Completed | 79 (16 treated IV; 63 treated SC) | 0.3, 0.6, or 1.0/IV/weekly/12 wk or 0.7, 1.0, 2.0, or 4.0/SC/weekly/12 weeks |
| HUPS252 (II) | Completed | 145 | 0.1 or 0.3 efalizumab or placebo/IV/weekly/8 weeks |
| ACD2142g (Ie) | Completed | 70 | 1.0 or 2.0/SC/12 weeks |
| ACD2058g (III) | Completed | 498 | 1.0 or 2.0 efalizumab or placebo/SC/weekly/two 12‑week courses |
| ACD2059g (III) | Completed | 597 | 1.0 or 2.0 efalizumab or placebo/SC/weekly/first 12‑week course 2.0 weekly or every other week or 4.0 efalizumab or placebo/SC/weekly/second 12‑week course |
| ACD2062g (III) | Completed | 775 (roll over from Studies ACD2058g, ACD2142g & previous XOMA Phase I/II trials) | 1.0 or 2.0/SC/weekly/12 weeks |
| ACD2243g (III) | Ongoing | 339 | 2.0/SC/weekly/12 weeks followed by 1.0/SC/weekly/up to 48 weeks |
| ACD2389g ^a^ (I) | Completed | 99 | 1.0/SC/every 6 weeks/two doses |
| ACD2390g (IIIb) | Completed | 556 | 1.0 efalizumab or placebo/SC/weekly/ 12 weeks |
| ACD2391g (IIIb) | Ongoing | Up to 556 who are eligible to roll over from Study ACD2390g | 1.0/SC/12 weeks with taper regimen |
| SC=subcutaneous.  ^a^ All subjects had moderate to severe plaque psoriasis except the healthy volunteers in Studies ACD2389g and ACD2617g. | | | |

| Table 1 (cont’d)  Efalizumab Clinical Studies in Psoriasis Program | | | |
| --- | --- | --- | --- |
| Study (Phase) | Status | Subjects ^a^ | Dose (mg/kg)/ Route/Frequency/Duration |
| ACD2600g (IIIb) | Ongoing | ~450 | 1.0 efalizumab or placebo/SC/weekly/ 12 weeks |
| ACD2601g (IIIb) | Ongoing | Up to 450 eligible subjects from Study ACD2600g | 1.0/SC/weekly/48 weeks |
| ACD2617g (I) | Ongoing | 60 | 1.0/SC/2× |
| SC=subcutaneous.  ^a^ All subjects had moderate to severe plaque psoriasis except the healthy volunteers in Studies ACD2389g and ACD2617g. | | | |

Findings from the Phase I, II, and III studies demonstrate that efalizumab is well tolerated, safe, and efficacious in subjects with moderate to severe plaque psoriasis at weekly doses of up to 2.0 mg/kg.

Safety experience for the Phase Ia-Id and Phase II studies is summarized in the Investigator Brochure.

Tables 2, 3, and 4 list the most common adverse events reported in the completed Phase III studies, ACD2058g, ACD2059g, and ACD2390g, respectively.

| Table 2  Study ACD2058g: Adverse Events Occurring in ≥5% of All Subjects during the Initial 12 Weeks of Treatment | | | | |
| --- | --- | --- | --- | --- |
|  |  | Efalizumab | |  |
| Adverse Event | Placebo  (n=169) | 1.0 mg/kg/wk  (n=162) | 2.0 mg/kg/wk  (n=165) | All Subjects  (n=496) |
| Total | 130 (76.9%) | 135 (83.3%) | 148 (89.7%) | 413 (83.3%) |
| Headache | 51 (30.2%) | 56 (34.6%) | 59 (35.8%) | 166 (33.5%) |
| Infection | 23 (13.6%) | 23 (14.2%) | 17 (10.3%) | 63 (12.7%) |
| Pain | 17 (10.1%) | 21 (13.0%) | 17 (10.3%) | 55 (11.1%) |
| Chills | 10 (5.9%) | 20 (12.3%) | 22 (13.3%) | 52 (10.5%) |
| Nausea | 16 (9.5%) | 14 (8.6%) | 21 (12.7%) | 51 (10.3%) |
| Asthenia | 17 (10.1%) | 16 (9.9%) | 12 (7.3%) | 45 (9.1%) |
| Fever | 9 (5.3%) | 12 (7.4%) | 18 (10.9%) | 39 (7.9%) |
| Diarrhea | 12 (7.1%) | 12 (7.4%) | 13 (7.9%) | 37 (7.5%) |
| Accidental injury | 6 (3.6%) | 17 (10.5%) | 12 (7.3%) | 35 (7.1%) |
| Pharyngitis | 15 (8.9%) | 9 (5.6%) | 10 (6.1%) | 34 (6.9%) |
| Rhinitis | 14 (8.3%) | 14 (8.6%) | 4 (2.4%) | 32 (6.5%) |
| Herpes simplex | 10 (5.9%) | 10 (6.2%) | 11 (6.7%) | 31 (6.3%) |
| Myalgia | 8 (4.7%) | 13 (8.0%) | 10 (6.1%) | 31 (6.3%) |
| Deafness ^a^ | 5 (3.0%) | 12 (7.4%) | 12 (7.3%) | 29 (5.8%) |
| Dizziness | 8 (4.7%) | 11 (6.8%) | 8 (4.8%) | 27 (5.4%) |
| ^a^ Adverse events coded to the term “deafness” were asymptomatic changes in audiogram thresholds for all but 2 subjects. | | | | |

| Table 3  Study ACD2059g: Adverse Events Occurring in ≥5% of All Subjects during the Initial 12 Weeks of Treatment | | | | |
| --- | --- | --- | --- | --- |
|  |  | Efalizumab | |  |
| Adverse Event | Placebo (n=122) | 1.0 mg/kg/wk (n=232) | 2.0 mg/kg/wk (n=243) | All Subjects (n=597) |
| Total | 90 (73.8%) | 199 (85.8%) | 207 (85.2%) | 495 (83.1%) |
| Headache | 29 (23.3%) | 71 (30.7%) | 93 (38.3%) | 193 (32.4%) |
| Infection | 19 (15.6%) | 27 (11.7%) | 43 (17.7%) | 89 (14.9%) |
| Nausea | 11 (9.0%) | 34 (14.7%) | 34 (14.0%) | 79 (13.3%) |
| Chills | 3 (2.5%) | 36 (15.6%) | 31 (12.8%) | 70 (11.7%) |
| Pain | 4 (3.3%) | 33 (14.3%) | 28 (11.5%) | 65 (10.9%) |
| Fever | 6 (4.9%) | 26 (11.2%) | 29 (11.9%) | 61 (10.2%) |
| Asthenia | 7 (5.7%) | 17 (7.4%) | 27 (11.1%) | 51 (8.6%) |
| Diarrhea | 11 (9.0%) | 15 (6.5%) | 17 (7.0%) | 43 (7.2%) |
| Myalgia | 5 (4.1%) | 16 (6.9%) | 22 (9.1%) | 43 (7.2%) |
| Arthralgia | 6 (4.9%) | 22 (9.5%) | 12 (4.9%) | 40 (6.7%) |
| Pharyngitis | 6 (4.9%) | 14 (6.0%) | 21 (8.6%) | 41 (6.9%) |
| Rhinitis | 8 (6.6%) | 18 (7.8%) | 13 (5.3%) | 39 (6.5%) |
| Peripheral edema | 5 (4.1%) | 14 (6.1%) | 11 (4.5%) | 30 (5.0%) |

Acute adverse events were defined as headache, chills, fever, nausea, or myalgia occurring within 48 hours of subcutaneous (SC) study drug administration. Acute adverse events occurred more frequently in the efalizumab groups than in the placebo group after the first two injections of study drug, but occurred with comparable frequency following subsequent injections.

The term “deafness” stands for any audiogram change without being associated with a subjective change in hearing. 1 patient in a phase 1 study experienced a unilateral hearing loss, which was reversible, and when assessed by a specialist, was thought not to be drug related.

As of 16 June 2003, 23 serious adverse events that occurred in Studies ACD2058g, ACD2059g, ACD2600g, ACD2601g, ACD2062g, ACD2243g, and ACD2390g and have been filed as Investigational New Drug (IND) safety reports: laryngospasm, thrombocytopenia (8 cases-see below), Legionella pneumonia, exfoliative erythroderma, elevated liver function test results, vertebral osteomyelitis, fever, squamous cell carcinoma, Hodgkin’s lymphoma, pneumonia, abscess of the left leg, surgical wound abscess, inflammatory arthritis, priapism, pustular psoriasis of Von Zumbusch, demyelinating process, adenopathy, allergic drug reaction, sinusitis, equilibrium disturbance, and eosinophilic pneumonitis.

In the combined safety database of 2762 patients, there were eight occurrences (0.3%) of thrombocytopenia of <52,000 cells per ul reported. Three of the eight patients were hospitalized for thrombocytopenia, including one patient with heavy uterine bleeding; all cases were consistent with an immune mediated thrombocytopenia. Antiplatelet antibody was evaluated in one patient and was found to be positive. Each case resulted in discontinuation of efalizumab. Based on available platelet count measurements, the onset of platelet decline was between 8 and 12 weeks after the first dose of efalizumab in 5 of the patients. Onset was more delayed in 3 patients, occurring as late as one year in 1 patient. In these cases, the platelet count nadirs occurred between 12 and 72 weeks after the first does of efalizumab. (This information is taken from the drug label.)

Four deaths have occurred that were determined by the investigators to be unrelated to study drug.

**Preliminary Findings from Mechanistic Studies**

We conducted an initial mechanism-of-action study for efalizumab as a sub-study within a larger, multicenter pharmacokinetics (PK) trial. At our site, 14 patients participated in the PK trial, and 13/14 agreed to co-participate in our mechanistic sub-study. The major analysis endpoint was at 8 weeks of treatment, as 8 weeks had served as the primary endpoint in earlier phase II trials. However, it has now become clear, that 12 weeks would have been a better primary endpoint, as later phase II and phase III trials have primarily done efficacy evaluations after 12 weeks of treatment with efalziumab.

During the mechanism-of-action (MOA) sub-study, various measures were made on circulating T-cells and biopsies of psoriatic skin lesions. On circulating T-cells, the expression of various adhesion and activation-related proteins was measured by flow cytometry (FACS) analysis. In collaboration with Sarah Bodary at Genentech, activation of T-cells at baseline (before treatment) and after 2 weeks of treatment was assessed using anti-CD3 antibodies to ligate the T-cell receptor (TCR) complex. Activation was assessed by induced expression of CD69 and CD25. In some cases activation was also assessed by measuring induced expression of cytokines (intracellular cytokine measures by FACS) or by cell proliferation assays. We also assessed cytokine-producing potential of circulating T-cells after PMA/ionomycin-induced activation along with induced expression of CD69, which was the activation control for these experiments.

FACS measures showed 1) CD11a and CD18 were down-regulated on T-cells, as had been expected from previous analyses. We developed a new assay to assess saturation of residual CD11a binding sites on T-cells which showed more complete saturation from weeks 2 to 8 than had been previously measured; 2) There was unanticipated down-regulation of the integrin VLA-4. Preliminary experiments done in collaboration with Tom Kupper's lab showed decreased binding of T-cells to fibronectin, a VLA-4 ligand. Data on more patients treated with efalizumab are required to confirm this finding prior to publication; 3) Many unrelated activation-controlling or activation related proteins were unexpectedly down-regulated. CD3 (ε-subunit) expression was unaffected by efalizumab, but decreased expression of TCR α/β subunits, CD4, CD8, CD2, and CD25 was measured at 2 weeks after starting treatment (and these decreases persisted through week 8); 4) T-cell activation using CD3 antibodies was markedly impaired (neither CD69 nor CD25 were up-regulated after CD3 ligation). More limited measures showed decreased production of cytokines or cell proliferation after efalizumab administration; 5) T-cell activation with PMA/ionomycin was essentially normal after efalizumab administration; 6) efalizumab treatment induced a lymphocytosis which, surprisingly, seemed to involve mainly an increase in circulating CD8+ memory T-lymphocytes.

The initial findings are very consistent in describing decreased expression of T-cell surface proteins and decreased activation of T-cells through the TCR complex, but the mechanisms associated with these findings have not been established. In addition, the reversibility of these effects following drug discontinuation (and CD11a re-expression) has not been addressed. In sum, FACS experiments show several novel effects of efalizumab on circulating T-cells that had not been previously appreciated. It is likely that efalizumab has a major effect on the activation potential of T-cells through the TCR complex, with the effects extending more broadly than "co-stimulation" blockade. Additional new experiments will be required to confirm and extend these preliminary observations (as well as to try to provide a biochemical explanation for decreased activation after CD3 ligation).

Effects of efalizumab on psoriasis disease activity, infiltration of skin lesions by T-cells (and other leukocytes), and expression of inflammation-related genes was assessed in skin biopsies obtained from 13 patients in the MOA sub-study. Six of 13 patients were judged to have a good response to efalizumab based on decreased epidermal hyperplasia and elimination of keratin 16 immunostaining in week 8 biopsies. On average, T-cells were reduced about 50% in week 8 biopsies from skin lesions. Quantitative RT-PCR measures of disease-related or inflammation-related genes showed consistent reductions in K16, IL-12 p40, γ-interferon, STAT-1, IL-8, and iNOS in good responders (by the histologic definition of response). Quantitative suppression of inflammation-related genes correlated better with response status than quantitative reductions in T-cells within lesions. To the extent that the group of inflammation-related genes forms a functionally-linked pathway, all measures were grouped into a "pathway score" according to a multivariate statistical method developed by Dr. Knut Wittkowski. By the same techniques, a composite clinical response score was constructed using a combination of histologic response measures and K16 gene expression. Composite clinical responses were much more highly correlated with an inflammation pathway score compared to a T-cell infiltration score.

Our overall analysis of results obtained with peripheral blood T-cells and psoriatic skin lesions suggests that one of the most important mechanistic effects of efalizumab may be suppression of T-cell activation, leading to decreased production of γ-interferon and other inflammatory products regulated downstream by γ-interferon/STAT1. As this view would significantly alter the current hypothesis that efalizumab improves psoriasis mainly by reducing T-cell trafficking into inflammatory skin sites, our new hypothesis would be significantly strengthened through the study of additional patients. In addition, the study of new patients would provide the opportunity to explore the mechanism by which T-cell activation is broadly affected by relatively selective targeting of a single cell surface integrin.

We have recently completed analysis of data from another study treating psoriasis patients with efalizumab (0.3mg/kg/wk for 8 weeks) (Xoma254) and observed that lesional CD11c+ dendritic cells are increased in psoriasis patients, and reduced in those who receive this therapy, especially in responders (K16 negative by immunohistochemistry). These CD11c+ DC produce iNOS and thus may be mediating inflammation.

**B) Hypotheses:**

Psoriasis is mediated through a type-1 pathway that can be affected by blocking CD11a.

1. CD11a blockade correlates with suppression of Type 1 inflammatory gene products;
2. Functional blockade of T-cell activation will persist only while LFA-1 is blocked/ down-regulated by Efalizumab.
3. T-cell activation through TCR complex will be blocked by efalizumab
4. Efalizumab decreases VLA-4 expression and this will have the functional consequence of blocking cell adhesion to fibronectin (a VLA-4 ligand).
5. **Aim:**

Primary:

- To determine whether improvement of target plaque under therapy with Efalizumab is correlated with changes in expression of defined inflammatory genes in the target plaque, and to characterize the contribution of defined inflammatory genes to the overall psoriatic inflammation process at different time points.

Secondary:

- To assess the correlation between functional CD11a blockade and response to treatment.
- To determine the mechanism of action of efalizumab in psoriasis focusing on blockade of important surface proteins during immune synapse formation (including TCR, LFA-1, VLA-4).
- To determine alterations of circulating leukocytes during and after efalizumab treatment.

1. **Primary outcomes:**
2. Clinical improvement of target lesions as measured by the **“Clinical Response Score”,** a single composite score based on quantitative measurement of epidermal acanthosis, qualitative expression of Keratin16 (histochemistry assessment) and quantitative measurement of K16 mRNA.
3. Measure genomic modulation by efalizumab real time RT-PCR for a set of pre-defined disease associated genes.
4. Characterization of inflammation through integration of quantitative expression of mRNAs encoding IL-12, gamma-interferon, STAT1, iNOS and IL-8 into the **“Type 1 pathway score”** (composite score derived by a novel multivariate analysis program).
5. Measure broad genomic modulation of psoriatic genes on the mRNA level using Affymetrix arrays in conjunction with cluster analysis programs at baseline and day 84 in circulating leukocytes and cells within psoriatic skin lesions.
6. **Secondary outcomes:**
7. Assessment of overall clinical response using the standard PASI score and a linear PASI score at weeks 6-24. A newer saturation assay will be performed to determine if clinical response is only maintained while functional blockade continues. Different conformational states of LFA-1 and their association with treatment will be assessed in both circulating cells and in tissue sections (depending on availability of reagents).
8. Assess the fraction of T-cells binding ICAM or fibronectin at week 2 compared to the fraction of T-cells binding these ligands at baseline (assessment of LFA-1 and VLA-4 integrin function).
9. *In vitro* assessment of T-cell activation after CD2 and CD3 ligation (with antibody) or TCR ligation (with superantigen) using assays that assess temporal events in the activation process (eg. calcium influx, CD69 induction, CD25, IL-2 induction, IL-2 receptor up-regulation, and cell proliferation). If calcium influx is blocked, we will explore signal transduction events that occur within the first two minutes of TCR ligation, eg., tryrosine phosphorylation of regulatory molecules such as ZAP-70 or the TCR zeta chain. We may also explore whether formation of an immune synapse is impacted by down-regulation of several protein subunits of the TCR complex following efalizumab administration.
10. To explore surface molecule expression and activatability of lesional T cells to establish cognate changes in circulating and lesional T cells in responders versus non-responders.
11. Characterize modulation of B cells, NK and NK-T cells, memory T-cells (central vs effector), monocytes, dendritic cells, neutrophils by FACS analysis during and after efalizumab treatment.
12. We will explore apoptosis of activated T cells in peripheral blood and at tissue level.
13. characterize CD11c+ DCs in the peripheral circulation and lesional tissue phenotypically and genomically. In-situ mediator production will also be determined by fluorescence microscopy. To test the hypothesis that the effect of efalizumab on DCs is an indirect effect via T cells, monocyte-derived DCs and autologous T cells will be co-cultured in the presence of efalizumab. How this biologic agent affects DC phenotype, activation, differentiation and maturation will be studied.
14. **Synopsis and brief description of the study**

This is an open-label study to investigate the mechanism of action of efalizumab (anti-CD11a). After a wash-out period from standard therapies, patients with moderate to severe psoriasis will be treated with subcutaneous injection of 1.0 mg/kg/week efalizumab for 24 weeks.

- Patients will be assessed by the PASI score and a modified, “linear PASI score”. Patients will also be categorized as “*responders*” and “*non-responders*” according to objective outcome criteria (**Clinical Response Score** and **Type-1 Pathway Score**). The primary goal is to study the cellular and genomic basis for therapeutic improvement or resistance to CD11a blockade.

At baseline, an index plaque will be selected for serial biopsy analysis. This plaque is located on the trunk, buttocks, or thigh (sensitive areas such as the face will not be biopsied). Punch biopsies will be performed from the index plaque at baseline (before treatment) and after 2, 6 and 12 weeks of treatment with efalizumab. The response to treatment at 12 weeks will be graded. Complete responders according to the **Clinical Response Score** will be defined as patients that reverse keratin 16 expression in conjunction with reversed acanthosis and normalized differentiation. The clinical responses will be graded from best to worst using a multivariate analysis technique that creates the “**Clinical Response Score**” based on a combination of epidermal thickness, the qualitative pattern of keratin 16 expression, and quantitative expression of keratin 16 mRNA. The stratification of clinical responses by this technique provides a measure of disease improvement that can be subsequently related to quantitative alterations in expression of other disease-related and inflammation-related genes.

The impact of CD11a blockade on a functionally linked pathway of inflammatory genes will be measured by the **Type-1 Pathway Score**. The genes included in this pathway are central to the activation of type 1 T-cells (IL-12, γ-IFN) and to genes regulating downstream inflammation (STAT1, IL-8, iNOS, MIG, etc). Because multiple genes are multiple independent variables, the **Type-1 Pathway Score** will serve as a novel multivariate analysis scoring tool. This allows us to compare “clinical” response outcomes **(Clinical Response Scores**) to modulation of type 1 inflammation-related genes **(Type-1 Pathway Score**). We will study the extent to which a response outcome is related to the number of residual T-cells in treated lesions versus the quantitative levels of γ-IFN mRNA (a direct measure of T-cell activation in plaques).

- Broad gene expression analysis in psoriatic skin lesions and blood at baseline, at the primary study endpoint (12 weeks of treatment) will be performed using a combination of gene arrays and real-time RT-PCR techniques.
- Cellular mechanisms associated with reductions in activation-controlling surface proteins on circulating and lesional T cells before and during efalizumab treatment will be studied. Previous work showed that T-cell activation is significantly impacted by efalizumab and several activation-controlling surface proteins are down-regulated during active treatment. Peripheral blood T-lymphocytes will be analysed at baseline, week 12 and in 2 week intervals after the last dose of efalizumab. This will include an analysis of dendritic cells in the peripheral circulation.
- Preliminary data demonstrated that VLA-4, an integrin unrelated to LFA-1, was down-regulated by efalizumab. Here, we will explore the functional consequences of VLA-4 modulation by efalizumab using functional binding assays with fibronectin as a ligand. We will determine whether this alteration is caused by reduced synthesis of VLA-4 or altered intracellular trafficking of integrin molecules. This will be in conjuction with Dr Thomas Kupper’s laboratory.
- Phenotype of cell suspensions of shave biopsies will be determined at baseline, week 2 and week 12, particularly to classify DC populations.

**G) Study design**

materials and methods

Subject Selection

All subjects volunteering for participation in the study will be screened using the following inclusion and exclusion criteria. Approximately 30 subjects with moderate to severe plaque psoriasis will be entered into the study.

Inclusion Criteria

Subjects must meet the following inclusion criteria to be eligible for study entry:

- Signed informed consent
- Plaque psoriasis covering >10% of total BSA
- Diagnosis of plaque psoriasis for at least 6 months
- PASI score >=12 (see Appendix A) or Linear PASI score of >= 8.0 at screening (see Appendix B).
- In the opinion of the investigator, candidate for systemic therapy for psoriasis:

A. Who has not been previously treated (naive to systemic treatment) OR

B. Who has had prior treatment with systemic therapy for psoriasis (e.g., PUVA, cyclosporine, corticosteroids, methotrexate, oral retinoids, mycophenolate mofetil (MMF), thioguanine, hydroxyurea, sirolimus, azathioprine, 6‑MP, etanercept)

- Body weight of <140 kg
- 18 to 75 years old. As the risk of Efalizumab in childhood is unknown, those < 18years will be excluded from the study.
- For women of childbearing potential or in men whose partners may become pregnant, willingness to use an acceptable method of contraception to prevent pregnancy for the duration of the study (while receiving study medication and 3 months following). Acceptable methods of contraception include use of a condom; abstinence; use by sexual partner of oral implantable or injectable contraceptives, IUD, female condom, diaphragm with spermicide, cervical cap; or a sterile sexual partner
- Willingness to hold sun exposure reasonably constant and to avoid use of tanning booths or other UV light sources during the study

Exclusion Criteria

Subjects who meet any of the following exclusion criteria are ineligible for study entry:

- Guttate, erythrodermic, or pustular psoriasis as sole or predominant form of psoriasis
- History of severe allergic or anaphylactic reactions to humanized monoclonal antibodies or fusion proteins that contain an Ig Fc region
- Clinically significant psoriasis flare during screening or on the first treatment day
- Treatment with efalizumab (anti-CD11a) within the last 12 months before enrollment
- Pregnancy or lactation. As the risk of Efalizumab in pregnancy is unknown, pregnant women will be excluded from the study.
- History of or ongoing uncontrolled bacterial, viral, fungal, or atypical mycobacterial infection
- History of active tuberculosis (TB) or currently undergoing treatment for TB. PPD testing or chest X-ray is required for high‑risk subjects (see Appendix D). Subjects with a positive PPD (not due to BCG vaccination) or chest X-ray will be excluded
- History of opportunistic infections (e.g., systemic fungal infections, parasites)
- Seropositivity for human immunodeficiency virus (HIV)
- Seropositivity for hepatitis B or C virus
- Hepatic enzymes >3 times the upper limits of normal (ULN)
- Diagnosis of hepatic cirrhosis, regardless of cause or severity
- WBC count <4000μL or >14,000/μL
- Serum creatinine >2 times the ULN
- Hospital admission for cardiac disease, stroke, or pulmonary disease within the last year
- Presence of malignancy within the past 5 years, including lymphoproliferative disorders. Subjects with a history of fully resolved basal cell or squamous cell skin cancer may be enrolled.
- History of substance abuse within the last 5 years
- Any medical condition that, in the judgment of the investigator, would jeopardize the subject’s safety following exposure to study drug

Note: Certain medications and vaccines may not be used for specified periods of time prior to enrollment or at any time during the treatment period. Please see text on page 21 and 22 regarding excluded therapies.

Study Treatment

Formulation

Efalizumab (Raptiva) is manufactured by Genentech. Efalizumab is supplied in single‑use glass vials containing 125 mg of sterile, pyrogen‑free, lyophilized drug product. When reconstituted with 1.3 mL of Sterile Water for Injection (SWFI), each vial contains efalizumab at a concentration of 100 mg/mL. The solution is clear. Reconstituted efalizumab is composed of efalizumab (125 mg), polysorbate 20, L‑histidine hydrochloride, sucrose, and SWFI, USP at a pH of 6.2.

For further details, see the Investigator Brochure.

Dosage, Administration, and Storage

Each subject will receive 24 weekly doses of 1.0 mg/kg study drug. Study drug will be administered by SC injection.

The following formula will be used to calculate dose volume:

| Dose volume (mL) = | Assigned dose (mg/kg) × body weight (kg) |
| --- | --- |
|  | 100 (mg/mL) |

A trained member of the research team at the research clinic will perform all injections for the first 12 weeks. Patients will be taught self-injection of efalizumab (as is accepted medical practice in the community), and will do so for weeks 13-24. Injection sites should be rotated weekly. Injection sites include the upper arms, buttocks, abdomen, and thighs.

Vials should be refrigerated at 2°C–8°C (36°F–46°F). Do not freeze or store at room temperature. However, non‑reconstituted product is stable at room temperature for up to 14 days, and reconstituted product is stable at room temperature for up to 8 hours, so small perturbations in storage are tolerable. After reconstitution, vials should be handled gently to avoid foaming. Do not vortex or agitate vigorously. All transfer procedures must adhere strictly to aseptic technique.

Reconstitution and study drug administration instructions are provided in Appendix E.

4.3.3 Dosage Modification

a. Change in Injection Volume

At any clinic visit during which study drug is administered, if a subject’s body weight has changed by ≥10% from the body weight used for the calculation of the previous injection volume, a new injection volume should be calculated based on the new body weight.

b. Missed Doses

Missed doses will not be replaced.

If a subject misses a dose of study drug (e.g., Treatment Day 7), dosing should resume with the next scheduled dose (e.g., Treatment Day 14).

Assessments during Treatment (Study Flowchart Appendix H)

All procedures should be completed prior to dosing.

Visit tolerance dates are +/-2 days from the schedule relative to Day 0.

- - - - 1. Day 0 (First Treatment Day)
- Review of inclusion and exclusion criteria
- Review and update of medical history for any interim events between screening and Day 0
- Complete physical examination, including dermatologic examination
- Vital signs (pre-dose; blood pressure while the subject is seated, pulse rate, and body temperature)
- Body weight and calculation of conditioning dose- faxed to pharmacy
- PASI score (Appendix A)
- Linear PASI Score (Appendix B)
- Psoriatic BSA (Appendix C)
- Medical photographs
- Review and recording of concomitant medications
- Skin biopsy (6mm punch biopsies of normal and psoriatic skin)
- Skin biopsy (optional small shave biopsies, approximately 1 cm by 1 cm, of normal and psoriatic skin)
- Research blood tests (see table Appendix F)
- Hematology: CBC with differential and platelet count
- Urine pregnancy test (predose; for women of childbearing potential only). If test results are positive, confirm findings with a serum pregnancy test. Do not administer study drug unless the serum pregnancy test result is negative.
- Administration of study drug (see Appendix E)
- Monitoring and recording of any adverse events following administration of study drug
- Subject Instruction Sheet

**b. Day 7 (Week 1)**

- Vital signs
- Body weight and dose calculation
- Review and recording of concomitant medications
- Monitoring and recording of adverse events
- Administration of study drug

**c.** **Day 14 (Week 2)**

- Vital signs
- Body weight and dose calculation
- PASI score and Linear PASI score, Psoriatic BSA
- Review and recording of concomitant medications
- Medical photographs
- Research blood
- Hematology: CBC with differential and platelet count
- Skin biopsy (6mm punch biopsies of psoriatic skin)
- Skin biopsy (optional small shave biopsies, approximately 1 cm by 1 cm, of normal and psoriatic skin)
- Monitoring and recording of adverse events
- Administration of study drug

d. Day 21 (Week 3)

- Vital signs
- Body weight and dose calculation
- Review and recording of concomitant medications
- Monitoring and recording of adverse events
- Administration of study drug

e. Day 28 (Week 4)

- Vital signs
- Body weight and dose calculation
- Review and recording of concomitant medications
- Urine pregnancy test
- Monitoring and recording of adverse events
- Administration of study drug
- Research blood

f. Day 35 (Week 5)

- Vital signs
- Body weight and dose calculation
- Review and recording of concomitant medications
- Monitoring and recording of adverse events
- Administration of study drug

g. Day 42 (Week 6)

- Vital signs
- Body weight and dose calculation
- Review and recording of concomitant medications
- PASI score and Linear PASI score, Psoriatic BSA
- Medical photographs
- Skin biopsy (6mm punch biopsy of psoriatic skin)
- Research Blood
- Hematology: CBC with differential and platelet count
- Monitoring and recording of adverse events
- Administration of study drug

h. Day 49 (Week 7)

- Vital signs
- Body weight and dose calculation
- Review and recording of concomitant medications
- Monitoring and recording of adverse events
- Administration of study drug

i. Day 56 (Week 8)

- Vital signs
- Body weight and dose calculation
- Review and recording of concomitant medications \
- Urine pregnancy test
- Monitoring and recording of adverse events
- Administration of study drug
- Research blood

j. Days 63, 70, and 77 (Week 9, 10, 11)

- Vital signs
- Body weight and dose calculation
- Review and recording of concomitant medications
- Monitoring and recording of adverse events
- Administration of study drug

k. Day 84 (Week 12)

- Complete physical examination, including dermatologic evaluation
- Vital signs
- PASI score and Linear PASI score, Psoriatic BSA
- Medical photographs
- Review and recording of concomitant medications
- Skin biopsy (6mm punch biopsy of psoriatic skin; an additional skin biopsy will be taken if the target lesion is not representative)
- Skin biopsy (optional small shave biopsies of psoriatic skin, approximately 1 cm by 1 cm, optional)
- Research blood
- Hematology: CBC with differential and platelet count
- Urine pregnancy test
- Monitoring and recording of adverse events
- Instruction of medication administration

**l. Days 112, 140, and 168 (Weeks 16, 20, 24)**

- Vital signs
- PASI score and Linear PASI Score, Psoriatic BSA
- Research blood-optional
- Hematology: CBC with differential and platelet count-if research blood drawn
- Biopsy of psoriatic skin (6mm punch biopsy) and medical photographs will be done when and if relapse should occur. Relapse is defined as loss of >=50% of PASI improvement compared to baseline. An additional biopsy and medical photographs could also be taken, if and when morphologically unusual lesions develop, even if the definition of relapse (>=50 loss of original PASI improvement) is not met.
- Review and recording of concomitant medications
- Monitoring and recording of adverse events
- Urine pregnancy test monthly
- Complete physical examination, including dermatologic evaluation at Day 168

Concomitant and excluded Therapies

Concomitant Therapy

- The concomitant psoriasis medications that may be used during the study (screening and treatment periods) are:
- Emollients (e.g., petrolatum or Eucerin cream or lotion)
- Tar or salicylic acid for scalp psoriasis
- Potency Group VI or VII topical corticosteroids in small amounts for psoriatic lesions on the face, hands, feet, groin, or axillae

Emollients, scalp preparations, and topical corticosteroids should not be used on psoriatic lesions on the day of a scheduled clinic visit when a PASI evaluation will be performed. If desired, these agents may be applied after the clinic visit has been completed.

Acetaminophen will be administered prior to the administration of the first three doses to reduce the incidence and severity of flu-like symptoms (i.e., headache, chills, fever, nausea, vomiting, and myalgia) sometimes seen following efalizumab administration. If acetaminophen is ineffective or if the subject is allergic to acetaminophen, ibuprofen can be administered at the discretion of the investigator. Alternatively, subjects may be pretreated with non-steroidal, non-prescription analgesics at the Investigator’s discretion. If a subject experiences an acute adverse event of nausea or vomiting, the subject may be treated with any approved anti‑emetic, as required.

If a subject experiences acute adverse events (defined as fever, headache, chills, nausea, vomiting, or myalgia on the day of or the 2 days following an injection of study drug), acetaminophen or other nonprescription, nonsteroidal analgesics are recommended as the first therapy. If these are ineffective, any approved analgesic may be prescribed at the discretion of the investigator. If a subject experiences nausea or vomiting, the subject may be treated with any approved anti‑emetic, as required.

If a subject experiences relapse of psoriasis after withdrawal from efalizumab, systemic or UV therapy may be used to the investigator’s discretion.

Treatment regimens of β‑blockers, angiotensin-converting enzyme (ACE) inhibitors, antimalarial drugs, quinidine, interferon, or lithium should be held constant, if possible, between Day –28 and Day 84. Addition or changes in these medications do not require discontinuation from the study.

Excluded Therapies

The following concomitant therapies are excluded for specified times prior to enrollment and throughout the treatment period:

- Systemic treatments for psoriasis (e.g., PUVA, cyclosporine, corticosteroids, methotrexate, oral retinoids, MMF, thioguanine, hydroxyurea, sirolimus, azathioprine, 6-MP, etanercept) (excluded from Day –28 of the screening period through Day 84)
- Immunosuppressive medications for any indication (excluded from Day –28 through Day 84)
- Live virus or bacteria vaccines, which include, but are not limited to, measles, mumps, rubella, polio, bacille Calmette‑Guerin (BCG), yellow fever, and TY21a typhoid (excluded from Day –14 through Day 84)
- Concomitant experimental drugs or treatments (excluded from Day –28 or five half-lives, whichever is longer, prior to Day 0 through Day 84)

If a subject receives one of the above medications during the screening period, the subject should not be included. If a subject receives one of these excluded concomitant therapies during the treatment period, the subject should not receive any additional doses of study drug and should be discontinued from the study.

The following additional concomitant therapies should be discontinued for at least 14 days prior to treatment:

- UVB phototherapy, tanning booths, or other nonprescription UV light sources
- Topical treatments for psoriasis (e.g., Potency Group I and II topical corticosteroids, calcipotriene, tazarotene, anthralin).
- Vaccines that do not contain live viruses or bacteria (e.g., flu, Pneumovax , tetanus)

These vaccines may not be effective when administered in the presence of therapeutic circulating levels of Efalizumab.

Instructions to Subjects

The following instructions should be given to subjects:

- All medications being used must be reported to the investigator.
- Donations of blood or blood products are not allowed during the study and for at least 3 months following the last injection of study drug.
- Treatment with immunotherapies such as killed virus vaccines, toxins, and allergy desensitization may not be effective during therapy with Efalizumab.

Subjects who require such vaccines (e.g., tetanus toxin booster, influenza vaccine) should be encouraged to schedule these at least 14 days prior to Day 0 or at least 3 months following the last injection of study drug.

- Vaccines that contain live organisms (e.g., BCG, measles) should not be administered in the presence of study drug (Day–14 through Day 84).
- If any new drugs are prescribed by any health care provider during a subject’s participation in the study, the subject should notify the prescribing physician of his or her participation in this study and notify the study staff of the new medications.
- Tanning beds, booths, or home UV light sources are prohibited from
  Day –14 through Day 84.
- If the subject experiences acute adverse events, such as headache, fever, chills, myalgia, nausea, or vomiting 12–48 hours following SC injection of study drug, the subject may be provided with the recommended therapy. Headache, fever, chills, or myalgia may be treated with acetaminophen or other nonprescription analgesic, if needed. Nausea or vomiting may be treated with an approved anti-emetic as needed. If the symptoms are severe, the subject should contact the investigator.
- Allergic reactions or hypersensitivity reactions such as hives or throat tightness should be reported immediately to the investigator. The subject should also seek immediate medical attention.
- Signs and symptoms of an infection should be promptly reported to the investigator.

See appendix G for a detailed patient information sheet taken from the drug label.

These instructions apply through Day 84. Subjects will receive a written instruction sheet on Day 0.

Compliance with Laws and Regulations

This study will be conducted according to the International Conference on Harmonization E6 Guideline for Good Clinical Practice (GCP) and any federal, national, and local requirements.

Subject Discontinuation

Subjects may withdraw or be withdrawn from the study, at any time. Every effort will be made to obtain information on subjects who discontinue from the study prematurely. The procedures and assessments to be performed at the end of treatment visit will be the same as those for the Day 84 visit.

Criteria for Discontinuation of Treatment

The criteria for discontinuation of treatment include the following:

- Pregnancy
- Administration of a live virus or bacteria vaccine
- Initiation of excluded systemic treatment for psoriasis
- Initiation of excluded immunosuppressive treatment for any indication
- Initiation of excluded experimental medication or other treatment
- Any medical condition (e.g., opportunistic infections, malignancies, immune complex disorders) that the investigator determines may jeopardize the subject’s safety if he or she continues in the study.

Study Discontinuation

Genentech has the right to terminate this study at any time. Reasons for terminating the study may include the following:

- The incidence or severity of adverse events in this or other studies indicates a potential health hazard to subjects.

1. **Statistical Model**

Preliminary data suggest that patients responding to efalizumab have lower expression levels of cytokines associated with activation of T-cells (IL-12, γ-IFN, IL-8, iNOS, and STAT1) than patients not responding. The response had been determined by the expression of K16 mRNA and epidermal thickness on day 56.

To correlate overall response with overall cytokine expression, response and cytokine expression were scored using a novel application of u-statistics to multivariate data. In short, the (response or cytokine expression) profile of each patient (A) is compared one-by-one with the profiles of all other patients (X_i_). If patient A has a higher observation for each of the (response or cytokine expression) variables than patient X_i_, the score of patient A is increased by one, if all X_i_ variables are lower, the score is decreased by one. If some variables have higher observations, while others have lower observations, the score of patient remains unchanged.

The response score correlated well with histological findings. Patients judged independently as complete responders were assigned scores between 2.5 and 5.5, partial responders were assigned scores between –2.0 and 0.5; non-responders between –5.5 and –3.5. Epidermal CD3^+^ T-cells on day 56 had a week correlation of .52 with the response score, while dermal CD3^+^ T-cells were virtually uncorrelated (r=0.12). The T-cell pathway score for both types of CD3^+^ T-cells was 0.38.

The correlation of u scores for individual cytokines on day 56 with the response score ranged from 0.18 (γ-IFN) to 0.66 (IL-12). The inflammation pathway score based on the expression of all five cytokines was 0.80. The correlation was smaller for all subsets, indicating that all cytokines contribute, as one would expect from cytokines on a single pathway. Eliminating a single variable from the pathway score had the smaller effects on the correlation for IL12 (-0.01) and γ-IFN (-0.03) than for IL-8 (-0.07), STAT1 (-0.19), and iNOS (-0.21), consistent with the hypothesis that the latter cytokines are further “downstream”.

Among the 12 patients in this study, three patients were neither superior nor inferior to any other patient and, thus, could not be assigned an informative score. Comparing the remaining four patients with a positive response score to the five patients with a negative response score by the pathway score yielded test statistic of 6.102 (χ^2^, 1 d.f.). While this is nominally significant (p = 0.0135, exact), it cannot be excluded that results based on a study where the smaller group has only four patients are confounded.

Since this is a mechanism of action study, where the consequences of false negative results (not continuing research on an important mechanism of action) are at least as severe as the consequences of false positive results (doing more research on the current drug, even if the mechanism of action should turn out to be different). Therefore, level (α) and power (1-β) need to be chosen so that β≤α for. Thus, to achieve the conventional 5% level, a power of at least 95% would be required. Assuming t the within group variation and correlation of the data observed above is representative for data in the proposed study, the above test statistic of 6.102 can be interpreted as the non-centrality parameter of the chi-square distribution with 1 df.

The non-centrality parameter required to guarantee 95% power at the 5% level (two-sided) is 14.37. In the previous study of twelve patients, three patients (25%) were uninformative, because their results were uniquely ambiguous. Thus, a total sample size of 14.37/6.102*9*1.25=27 per group would be required to detect an effect as large as seen in the previous study with 95% power at the 5% level (two-sided). It is common for up to 10% of the enrolled patients to not complete the entire treatment protocol. Thus, we plan to enroll approximately 30 patients to allow for this possibility.

While we have chosen the expression of cytokines at day 84 as the primary outcome, we will also explore whether changes in cytokine expression at different points in time may even be more closely correlated with response.

As the mechanism of action is presently unknown, it cannot be determined, whether (a) differences in expression, (b) ratios in expression, or (c) ratios in expression above the expression in non-lesional skin are the most relevant parameters to measure changes in cytokine activity. Therefore, we will use a non-parametric approach, again based on u-statistics. In brief, we will consider a change in patient A larger than in patient B, if patient A has higher expression than patient B at the first and a lower expression than patient B at the second time point. If for neither patient the pre-post expression interval is included in the other patient, this pair of patients will not affect the computation of these patients’ u-scores. Replacing the set of cytokine levels with a set of cytokine changes and selecting for each cytokine a different time interval will allow us to explore the hypothesis that cytokines further downstream contribute earlier to the activity along the pathway.

11. Study Subjects:

Adults with plaque psoriasis covering > 10% of body surface area (BSA) and a PASI score of > 12.0 or linear PASI score >=8 at screening who are candidates for systemic psoriasis therapy, will be entered into the study. A subject is considered a candidate for systemic therapy if assessed by a clinician as requiring systemic therapy (e.g., psoralen with UV light [PUVA], cyclosporine, corticosteroids, methotrexate, oral retinoids, mycophenolate mofetil [MMF], thioguanine, hydroxyurea, sirolimus, azathioprine, 6-MP, etanercept) to control psoriasis, whether or not that subject has a history of receiving systemic therapy. Approximately 30 subjects will be enrolled.

**12. Recruitment and Consent Procedures:**

Subjects are recruited from the patient populations of The Rockefeller University, the New York Hospital, and general communities in which these institutions are located. Eligible patients selected will be of either sex in approximately equal numbers. As in all our protocols, individuals will be selected without regard to race or ethnicity.

13. Risks and Alternative Methods:

The risks to treatment with Efalizumab are outlined in a previous section. As with any medication, there are always unknown risks. Another theoretical risk is a possible allergic reaction. There are minor risks to skin biopsy (infection, bleeding, a small scar) and to phlebotomy from peripheral veins (bleeding, bruising). The risks of Efalizumab treatment in pregnancy or during lactiation are unknown.

Other treatments are available for the treatment of psoriasis, but each alternative (phototherapy, photochemotherapy, methotrexate, etretinate) have a therapeutic index, which precludes long-term treatment. In contrast, Efalizumab offers the potential of a treatment with reduced toxicity and with highly selective effects on cells mediating this disease.

**14. Data and Safety Monitoring Plan:**

This project entails moderate risk to subjects. Monitoring will be conducted a) by the Principal Investigator (PI) in consultation with the Clinical Research Safety Officer of the ACCTS and b) by the PI and the Rockefeller University Institutional Review Board (IRB), through annual reports of progress and by immediate notification of adverse events by the PI to the IRB, the ACCTS, and any federal regulatory agency (e.g. FDA) when appropriate or required.

The Rockefeller University Clinical Research Office will conduct spot audits, “for cause” audits, and reviews, upon the request of the investigators.

**Safety review and monitoring:**

All laboratory tests will be reviewed and signed by the investigator on a timely basis. MSKCC, which is a CLEP certified laboratory, will be used. Only lab results obtained from under CLEP certified conditions will be shared with patients.

Adverse events (AEs) are compiled for each patient, for each study, and on a weekly basis in a central book in the lab. Compiled data is verified using source documents. Furthermore, AE’s are reviewed on a monthly basis and tracked. Minutes will be kept of the monthly review.

Study monitoring will occur on a regular basis. Charts will be reviewed for completeness as source documents, e.g. grading and attribution of all AEs, start/stop information for all AEs, confirmation of study eligibility, up-to-date informed consent, submission of all amendments, etc., by the study coordinator. A log will document the occurrence of regular monitoring activities.

The study coordinator, or investigator, manages protocol-specific data on paper forms organized by patient number and/or study. These are kept in a secure, double locked area of the lab. All lab personnel that work with patient data or samples have passed human subject protection courses and will keep data confidential in designated laboratory files. Data are not recorded in computerized spreadsheets, except for final analysis of data. Since Microsoft excel is often used for data analysis, only the primary “sheet” is used and data cannot be copied to another sheet. Data entries are certified against primary source documents in the lab.

**Adverse Events (AEs)**

The Common Toxicity Criteria (CTC) Grading Scale v3.0 (<http://ctep.cancer.gov/forms/CTCAEv3.pdf>), a standard and widely used instrument, will be used to define, grade and attribute adverse events (AEs). Any AEs encountered during the study will be recorded, and AEs will be reported as outlined in the table below.

| **Type of AE** | **Report to:** | **Timeframe for reporting** | **Web Reference** |
| --- | --- | --- | --- |
| All AEs | RU IRB | Annually | <http://clinfo.rockefeller.edu/irb/irb-ind.htm>  Procedures Section 17 |
| Unexpected AEs > grade 2; Serious AEs | RU IRB | Within 2 working days* | <http://clinfo.rockefeller.edu/irb/irb-ind.htm>  Procedures Section 17 |
| Both serious and unexpected AEs | FDA, Sponsor | As soon as possible (phone, fax writing), but within 7 days* | <http://www.accessdata.fda.gov/scripts/cdrh/cfdocs/cfcfr/CFRSearch.cfm?fr=312.32> |

* time since discovery

All AEs will be reported directly to The Rockefeller University IRB and the ACCTS both orally at the annual meetings and in writing as an annual report in the protocol. The annual summary will include:

1-All AEs will be summarized in tabular form; including grade for all AEs, attribution information for grade 3 and 4, and dose reduction information where appropriate.

2-Total enrollment, demographics of enrollment, protocol violations current status of subjects (completed, ongoing, dropout), reasons for attrition, and number of subjects reaching primary endpoints.

3-Available research data, especially any that might provide evidence of early proof or dis-proof of the hypothesis.

Unexpected AEs (of grade 2 or greater severity) and any serious adverse event (SAE) will be reported to the **IRB** within 2 working days of discovery as directed by The Rockefeller University IRB Procedures Manual version date 2/3/07.

Unexpected AEs (of grade 2 or greater severity) and any serious adverse event (SAE) will be reported to the **FDA** and **Sponsor** as soon as possible after discovery by telephone, fax or writing, but within 7 days, and with a full written report within additional 8 calendar days. This conforms to standard good clinical practice (GCP) guidelines (ICH Tripartite Guideline. Clinical Safety Data Management: Definitions and Standards for Expedited Reporting, Part B, no 1)

Any IND Safety Reports received from the Sponsor will be filed with the IRB promptly.

Any pregnancy that occurs during the study, although not itself an SAE, should be recorded as an SAE and expeditiously reported to the Sponsor to facilitate outcome follow-up. Abortion, whether it be accidental, therapeutic, or spontaneous, should be reported as an SAE. Similarly, any congenital anomaly/birth defect in a child born to a subject exposed to the investigational treatment should be reported as an SAE.

A female subject must be instructed to stop taking study drug and immediately inform the investigator if she becomes pregnant during the study. Pregnancies beginning within 90 days after the completion of the last dose of study drug must also be reported to the investigator. The Investigator should counsel the subject, discussing the risks of continuing with the pregnancy and the possible effects on the fetus. Monitoring of the subject should continue until conclusion of the pregnancy. Pregnancy occurring in the partner of a subject participating in the study should also be reported to the investigator and Sponsor.

**15. Procedures to Minimize risks:**

Safety will be assessed through monitoring of all adverse events that occur following the first study drug treatment on Day 0. Subjects will be instructed about possible acute adverse events and first‑dose effects. Patients will be given detailed written instructions (see appendix G). Safety assessments will include routine laboratory tests (hematology, chemistry, urinalysis, and urine pregnancy tests, if appropriate). Periodic complete dermatologic examinations will be performed with careful surveillance for skin cancer and herpetic infections. In addition, a summary will be produced for each of the following:

- Serious adverse events
- Events leading to withdrawal from treatment
- Events judged by the investigator to be related to study drug
- All events by severity
- Acute adverse events
- Infections
- Events potentially indicative of hypersensitivity

Study Medication Accountability

All study drug required for completion of this study will be provided by Genentech. The recipient will acknowledge receipt of the drug by returning the INDRR-1 form indicating shipment content and condition. Damaged supplies will be replaced.

Accurate records of all study drug dispensed from and returned to the study site should be recorded on the Drug Inventory Log.

All partially used or empty containers should be disposed of at the study site according to institutional standard operating procedure. Return unopened, expired, or unused study drug with the Inventory of Returned Clinical Material form as directed by Genentech.

Disclosure of Data

Upon the subject’s permission, medical information may be given to his or her personal physician or other appropriate medical personnel responsible for his or her welfare.

Data generated by this study must be available for inspection upon request by representatives of the U.S. FDA, national and local health authorities, Genentech (and its licensees and joint developers), and the IRB/EC for each study site, if appropriate.

Retention of Records

U.S. FDA regulations (21 CFR §312.62[c]) require that records and documents pertaining to the conduct of this study and the distribution of investigational drug, including CRFs, consent forms, laboratory test results, and medication inventory records, must be retained by the Principal Investigator for 2 years after marketing application approval. If no application is filed, these records must be kept 2 years after the study is discontinued and the U.S. FDA and the applicable national and local health authorities are notified. Genentech will notify the Principal Investigator of these events.

For studies conducted outside the United States under a U.S. IND, the Principal Investigator must comply with U.S. FDA IND regulations and with those of the relevant national and local health authorities.

**16. Potential Benefits:**

For the individual patient, the major benefit that could be derived from this study is the complete clearing of psoriasis, without the need for alternative therapy. Benefits to society include the possibility that the study medication may prove to be more efficacious and have a superior therapeutic index than currently available anti-psoriatic therapies.

**17. Risk to Benefit Ratio -** favors participating in study

**18. Compensation:**

Patients will receive free care of their psoriasis (free drug but not free emollients).

REFERENCES

Bauer R. Pharmacokinetics of subcutaneously administered hu1124 in psoriasis subjects. Berkeley (CA):XOMA LTD.: 6 March 2000 Report No.: FR 104.

Bauer RJ, Dedrick RL, White ML, Murray MJ, Garovoy MR. Population pharmacokinetics and pharmacodynamics of the anti-CD11a antibody hu1124 in human subjects with psoriasis. J Pharmacokinetic Biopharm 1999;27:397–420.

Benjamin RJ, Qin SX, Wise MP, Cobbold SP, Waldmann H. Mechanisms of monoclonal antibody‑facilitated tolerance induction: a possible role for the CD4 (L3T4) and CD11a (LFA‑1) molecules in self‑non‑self discrimination. Eur J Immunol 1988;18:1079–88.

Cavazzana‑Calvo M, Sarnacki S, Haddad E, De Coene C, Calise D, Yvon E, et al. Prevention of bone marrow and cardiac graft rejection in an H‑2 haplotype disparate mouse combination by an anti‑LFA‑1 antibody. Transplantation 1995;59:1576–82.

Cobbold SP, Rebello PR, Davies HF, Friend PJ, Clark MR. A simple method for measuring patient anti‑globulin responses against isotypic or idiotypic determinants. J Immunol Methods 1990;127:19–24.

Dustin ML, Springer TA. Lymphocyte function‑associated antigen‑1 (LFA‑1) interaction with intercellular adhesion molecule‑1 (ICAM‑1) is one of at least three mechanisms for lymphocyte adhesion to cultured endothelial cells. J Cell Biol 1988;107:321–31.

Dustin ML, Springer TA. Role of lymphocyte adhesion receptors in transient interactions and cell locomotion [review]. Annu Rev Immunol 1991;9:27–66.

Fischer A, Durandy A, Sterkers G, Griscelli C. Role of the LFA‑1 molecule in cellular interactions required for antibody production in humans. J Immunol 1986;136:3198–203.

Gordon EJ, Myers KJ, Dougherty JP, Rosen H, Ron Y. Both efalizumab (LFA‑1) and anti‑CD11b (MAC‑1) therapy delay the onset and diminish the severity of experimental autoimmune encephalomyelitis. J Neuroimmunol 1995;62:153–60.

Gottlieb A, Krueger JG, Bright R, Ling M, Lebwohl M, Kang S, et al. Effects of administration of a single dose of a humanized monoclonal antibody to CD11a on the immunobiology and clinical activity of psoriasis. J Am Acad Dermatol 2000;42(3):428–38.

He Y, Mellon J, Apte R, Niederkorn JY. Effect of LFA‑1 and ICAM‑1 antibody treatment on murine corneal allograft survival. Invest Ophthalmol Vis Sci 1994;35:3218–25.

Isobe M, Yagita H, Okumura K, Ihara A. Specific acceptance of cardiac allograft after treatment with antibodies to ICAM‑1 and LFA‑1. Science 1992;255:1125–7.

Khazaeli MB, Conry RM, LoBuglio AF. Human immune response to monoclonal antibodies. J Immunother 1994;15:42–52.

Krensky AM, Sanchez‑Madrid F, Robbins E, Nagy JA, Springer TA, Burakoff SJ. The functional significance, distribution, and structure of LFA‑1, LFA‑2, and LFA‑3: cell surface antigens associated with CTL‑target interactions. J Immunol 1983;131:611–6.

Kuypers TW, Roos D. Leukocyte membrane adhesion proteins LFA‑1, CR3 and p150,95: a review of functional and regulatory aspects [review]. Res Immunol 1989;140:461–86.

Nakakura EK, McCabe SM, Zheng B, Shorthouse RA, Scheiner TM, Blank G, et al. Potent and effective prolongation by anti‑LFA‑1 monoclonal antibody monotherapy of non‑primarily vascularized heart allograft survival in mice without T-cell depletion. Transplantation 1993;55:412–7.

Nickoloff BJ, Griffiths CE, Barker JN. The role of adhesion molecules, chemotactic factors, and cytokines in inflammatory and neoplastic skin disease–1990 update [review]. J Invest Dermatol 1990;94(6 Suppl):151S–7S.

Nishihara M, Gotoh M, Fukuzaki T, Ohta Y, Monden M, Yagita H, et al. Potent immunosuppressive effect of anti‑LFA‑1 monoclonal antibody on islet allograft rejection. Transplant Proc 1995;27:372.

Springer TA, Dustin ML, Kishimoto TK, Marlin SD. The lymphocyte function‑associated LFA‑1, CD2, and LFA‑3 molecules: cell adhesion receptors of the immune system [review]. Annu Rev Immunol 1987;5:223–52.

Talento A, Nguyen M, Blake T, Sirotina A, Fioravanti C, Burkholder D, et al. A single administration of LFA‑1 antibody confers prolonged allograft survival. Transplantation 1993;55:418–22.

Tanaka Y, Takahashi A, Arai I, Inoue T, Higuchi S, Otomo S, et al. Prolonged inhibition of an antigen‑specific IgE response in vivo by monoclonal antibody against lymphocyte function‑associated antigen‑1. Eur J Immunol 1995; 25:1555–8.

van Dijken PJ, Ghayur T, Mauch P, Down J, Burakoff SJ, Ferrara JL, et al. Evidence that anti‑LFA‑1 in vivo improves engraftment and survival after allogeneic bone marrow transplantation. Transplantation 1990;49:882–6.

Werther WA, Gonzalez T, O’Connor SJ, McCabe S, Chan B, Hotaling T, et al. Humanization of an anti‑lymphocyte function‑associated antigen (LFA)‑1 monoclonal antibody and reengineering of the humanized antibody for binding to rhesus LFA‑1. J Immunol 1996;157:4986–95.

**APPENDIX A**

**Psoriasis Area and Severity Index (PASI)**

Evaluator Requirements

 The evaluator must be a dermatologist or an experienced health care provider.

 The evaluator must have attended the Investigator meeting or have been trained by someone who attended the Investigator meeting.

 The same evaluator should determine all PASIs for any individual subject throughout the trial.

A backup evaluator is only allowed in case of emergency or special situation when the designated evaluator is unable to perform the evaluation. The backup evaluator must be a dermatologist or an experienced health care provider and have attended the Investigator meeting or have been trained by someone who attended the Investigator meeting.

General Instructions

1. For each area of the body, rate erythema, scaling, and thickness for the **average** plaque or the overall condition of plaques in that region.

2. Rate plaques as they are actually seen on the day of examination. Do not rate in comparison with baseline condition.

PASI Scoring

PASI scores can range from 0 to 72. Dermatologic disease severity is to be scored as follows:

Body Areas

Four main body areas will be assessed, the head (h), the trunk (t), the upper extremities (u), and the lower extremities (l) corresponding to 10%, 30%, 20%, and 40% of the total body surface area (BSA), respectively.

APPENDIX A (cont’d)

Psoriasis Area and Severity Index (PASI)

The area of psoriatic involvement for each body area (Ah, At, Au, Al) will be assigned a numerical value according to degree of involvement as follows:

0 = no involvement
1 = <10% involvement
2 = 10% to <30% involvement
3 = 30% to <50% involvement
4 = 50% to <70% involvement
5 = 70% to <90% involvement
6 = 90% to 100% involvement

Conventions for estimating BSA include the following:

 Include only currently active disease in affected area:

For small, scattered lesions do not include the skin between the lesions in the estimate;

For a centrally cleared plaque, count only area of inflamed outer ring;

Do not include residual hyperpigmentation, hypopigmentation, pigmented macules, or diffuse slight pink coloration

 Assignments for the following body segments are:

Neck: include with the head
Buttocks: include with the lower extremities
Axillae: include with trunk
Genitals: include with the trunk
Separation of trunk and lower extremities: the inguinal canal separates the trunk and legs anteriorly

Psoriatic Lesion Signs

In addition, the severity of the psoriatic lesions in three main signs—erythema (E), thickness (T), and scaling (S)—will be assessed for each body area according to a scale (0–4) in which 0 represents a complete lack of cutaneous involvement and 4 represents the most severe possible involvement.

APPENDIX A (cont’d)

Psoriasis Area and Severity Index (PASI)

|  | Erythema a | Scaling | Thickness |
| --- | --- | --- | --- |
| 0=none | No redness | No scaling | No elevation over normal skin |
| 1=slight | Faint redness | Fine scale partially covering lesions | Slight but definite elevation, typically edges indistinct or sloped |
| 2=moderate | Red coloration | Fine to coarse scale covering most of all of the lesions | Moderate elevation with rough or sloped edges |
| 3=severe | Very or bright red coloration | Coarse, non-tenacious scale predominates covering most or all of the lesions | Marked elevation typically with hard or sharp edges |
| 4=very severe | Extreme red coloration; dusky to deep red coloration | Coarse, thick, tenacious scale over most or all lesions; rough surface | Very marked elevation typically with hard sharp edges |
| a Does not include residual hyperpigmentation, hypopigmentation, pigmented macules, or diffuse slight pink coloration as erythema. | | | |

APPENDIX A (cont’d)

Psoriasis Area and Severity Index (PASI)

Calculating PASI

To calculate the PASI, the sum of the severity rating for the three main signs will be multiplied with the numerical value of the area affected and with the various percentages of the four body areas. These values will then be added to complete the formula as follows:

| PASI = | 0.1 (Eh + Th + Sh) Ah + 0.3 (Et + Tt + St) At +  0.2 (Eu + Tu + Su) Au + 0.4 (El + Tl + Sl) Al |
| --- | --- |

| Row |  | Head | Trunk | | Upper Limbs | Lower Limbs | |  |
| --- | --- | --- | --- | --- | --- | --- | --- | --- |
| 1 | Erythema a |  |  | |  |  | |  |
| 2 | Thickness a |  |  | |  |  | |  |
| 3 | Scaling a |  |  | |  |  | |  |
| 4 | Total each column |  |  | |  |  | |  |
| 5 | Degree of involvement b |  |  | |  |  | |  |
| 6 | Multiply Row 4 by Row 5 |  |  | |  |  | |  |
| 7 |  | × 0.10 | × 0.30 | | × 0.20 | × 0.40 | |  |
| 8 | Multiply Row 6 by Row 7 |  |  | |  |  | |  |
| 9 | Total PASI (add together each column from Row 8) | | |  | | |  | |
| a Rank severity of psoriatic lesions: 0=none, 1=slight, 2=moderate, 3=severe, 4=very severe.  b Rank area of psoriatic involvement: 0=none, 1= <10%, 2=10% to <30%, 3=30% to <50%, 4=50% to <70%, 5=70% to <90%, 6=90% to 100%. | | | | | | | | |

APPENDIX B

LINEAR PASI

| Row |  | Head | Trunk | | Upper Limbs | Lower Limbs |  |
| --- | --- | --- | --- | --- | --- | --- | --- |
| 1 | Erythema a |  |  | |  |  |  |
| 2 | Thickness a |  |  | |  |  |  |
| 3 | Scaling a |  |  | |  |  |  |
| 4 | Total each column |  |  | |  |  |  |
| 5 | % of involvement |  |  | |  |  |  |
| 6 | Multiply row 4 by 5 |  |  | |  |  |  |
| 7 | Factor | 0.6 | 1.8 | | 1.2 | 2.4 |  |
| 8 | Multiply Row 6 by Row 7 |  |  | |  |  |  |
| 9 | Total PASI (add together each column from Row 8) | | |  | | |  |
| a Rank severity of psoriatic lesions: 0=none, 1=slight, 2=moderate, 3=severe, 4=very severe | | | | | | | |

This worksheet is a modification of a conventional PASI. Scaling, erythema, and thickness are determined in the same fashion and using the same descriptors as in the conventional PASI. The modification here is that the actual percentage involvement of head, trunk, and limbs is recorded so that increases or decreases in surface extent impact upon the PASI score in a 1:1 (or linear) fashion. For example, a decrease in surface involvement by 50% (but residual lesions have same erythema, thickness, and scale as baseline) would produce a 50% reduction in the linear PASI measure. The scale has been adjusted (by factors in row 7) so that the scale produces a measure from 0-72, as does the conventional PASI measure.

APPENDIX C

Psoriatic Body Surface Area (BSA)

The percentage of total body surface area (BSA) affected by psoriasis is to be calculated using the following table.

| Body Segment | % of Total BSA | Anterior Sites Affected  (% of Total BSA) a | Posterior Sites Affected  (% of Total BSA) a | Row Percentage Total |
| --- | --- | --- | --- | --- |
| Head | 10% | __________ + __________ | | ___ % |
| Trunk | 30% | __________ + __________ | | ___ % |
| Upper limbs | 20% | __________ + __________ | | ___ % |
| Lower limbs | 40% | __________ + __________ | | ___ % |
| Total | 100% |  | | ___ % |
| a Percentage of body surface affected by psoriasis (total of all affected segments). | | | | |

The first column of the table lists the four segments of the body (see Appendix A, Psoriasis Area and Severity Index, for assignment of body segments). The second column lists the approximate percentage of the entire body area represented by each segment. This percentage is to serve as a reference and represents the maximal area that can be reported in the fifth column. Using the “rule of palm” (1% BSA = palm to first interphalangeal joint), Columns 3 and 4 are to be determined. The rule of palm reference is determined based on the size of the subject’s palm and not the evaluator’s palm. Note: The percentage of anterior portion and percentage of posterior portion affected by psoriasis are to be determined as percentages of the total BSA. Column 5 is used to sum the totals of the four body segments to calculate the total BSA.

**APPENDIX D**

Tuberculosis (TB) Assessment

Tuberculin skin testing (purified protein derivative [PPD]) or a chest X-ray will be performed for the following high‑risk individuals:

 Individuals having close contact with individuals with active TB

 Individuals with a history of injection drug abuse

 Individuals with insulin‑dependent diabetes mellitus, silicosis, post‑gastrectomy, or jejunoileal bypass

 Individuals on prolonged (>3 months) high‑dose (systemic) corticosteroids (including inhalers) or other immunosuppressive therapies (e.g., methotrexate, cyclosporine, prednisone)

 Individuals with a body weight <90% of ideal body weight

 Individuals who live in institutional (high‑congregate) settings or shelters

 Individuals who are foreign‑born from Asia, Africa, or Latin America

 Individuals who are healthcare workers

Individuals with a history of bacille Calmette‑Guerin vaccination are required to have a chest X‑ray instead of PPD or to be able to document a negative chest X‑ray within the last year. The PPD skin test must be read between 48 and 72 hours.

Individuals with a newly positive TB skin test or chest X-ray are not eligible for enrollment. Individuals with an abnormal chest X‑ray showing fibrotic lesions consistent with previous TB are ineligible.

APPENDIX E

Study Drug Reconstitution and Administration

Study Drug Shipping and Packaging

The kits shipped to the study site will contain six vials of study drug. During the study each kit should be used for only 1 subject, but each subject will require more than one kit. Each vial will contain ~125 mg of study drug (efalizumab). If the packaging is damaged upon receipt or any unusual attributes or appearance of the study kits is observed, the kits should not be used and the problem should be immediately reported.

General Information and Instructions

 Vials should be refrigerated at 2^0^C–8^0^C (36^0^F–46^0^F). Do not freeze or store at room temperature. After reconstitution, vials should be handled gently to avoid foaming. Do not vortex. All transfer procedures must strictly adhere to aseptic technique.

 For safety, diphenhydramine hydrochloride for injection or another comparable antihistamine and epinephrine 1:1000 or 1:10,000 must be available at the site.

Reconstitution

1. Using an alcohol swab, wipe the rubber stopper of the Sterile Water for Injection (SWFI) and each vial of lyophilized study drug.

2. Using a 3‑cc syringe with 25‑gauge needle, withdraw 1.3 mL of sterile water from the SWFI vial.

3. Slowly withdraw the needle from the SWFI vial, being careful not to put pressure on the syringe plunger, and put the vial of SWFI down.

4. Leaving the vial of study drug on a firm surface, slowly puncture the rubber seal with the needle and syringe filled with SWFI.

5. Gently push down on the plunger to release the entire 1.3 mL of SWFI into the vial of lyophilized study drug.

6. Gently swirl the contents of the vial until contents have dissolved. Wait 5 minutes for the contents to completely dissolve. Do not shake the vial or “vortex” the contents.

7. Slowly withdraw the needle from the vial of study drug.

APPENDIX E (cont’d)

Study Drug Reconstitution and Administration

8. If more than one vial of study drug is required, repeat Steps 2–6.

9. Using a new alcohol swab, wipe the rubber stopper of the vial of reconstituted study drug.

10. Hold the vial of study drug upside down and insert a **3‑cc syringe** with a **25‑gauge needle** to retrieve the reconstituted drug product solution. If the injection volume is <1.0 mL, a 1‑cc syringe should be used in place of the 3‑cc syringe for the injection.

11. Remove the syringe and needle from the vial. To remove air trapped in between the needle and the drug, keep the needle pointed up and gently apply pressure to the plunger until a small amount of drug comes out of the needle.

12. If more than one vial of study drug is required per dose (the injection volume is >1.3 mL), repeat Steps 9 and 10.

Administration of Study Drug

 Administer by subcutaneous injection in the upper arm, thigh, buttock, or abdomen. Injections should be rotated systematically among the sites.

 No more than 1.5 cc of test article should be administered per injection; therefore, some subjects may require two or more injections to deliver the assigned dosage. If more than one injection is required, the test article should be divided equally among the syringes.

**APPENDIX F**

**Research Blood Tests**

1. **IMMUNOHISTOCHEMISTRY AND IMAGE ANALYSIS**

Tissue sections will be stained with haematoxylin and eosin (H&E) and with purified mouse anti-human monoclonal antibodies to K16, CD3, CD8 and CD103. Biotin-labeled horse anti-mouse antibody will be used for amplification, with avidin-biotin complex and developed with chromogen 3-amino-9-ethylcarbazole. Epidermal thickness measures will be computed using National Institute of Health software (NIH Image 6.1), and positive cells will be counted manually using computer-assisted image analysis. Conformational states of LFA-1 will be analyzed where there are suitable antibodies available for immunohistochemistry.

1. **RT-PCR ANALYSIS**

The PAXgene Blood RNA System is a commercially available integrated and standardized system for the collection and stabiization of whole blood specimens and isolation of cellular RNA. As an alternative, CPT tubes may also be used for this purpose. We will collect blood samples at time points indicated in Appendix I. Genes of interest include inflammatory genes and those responsible for the phenotype of psoriasis (γ-IFN, TNF-α, STAT-1, iNOS, IL-8, MIG, IP-10, IL-19, IL-20, IL-23); those representative of T-cell activation (IFN-γ, IL-12, CD25); dendritic cell activation (CD80, IL-12); keratinocyte proliferation (Keratin 16); Type 1 or Type 2 T-cell immune deviation (γ-IFN , IL-4, IL-10, IL-11) and new genes discovered from gene chip data (ENRAGE, MMP). Other genes may be studied as they are discovered or their relevance is newly appreciated.

RNA will be extracted from tissues frozen in liquid nitrogen using the RNeasy Mini Kit. The primers and probes for the TaqMan RT-PCR assays for chosen genes have been generated using the Primer Express algorithm, version 1.0, using published genetic sequences (NCBI-PubMed) for each gene. The RT-PCR reaction will be performed using EZ PCR Core Reagents according to the manufacturer's directions. The samples will be amplified and quantified on an Applied Biosystems PRISM 7700 using the following thermal cycler conditions: 2 min @ 50 °C; 30 min @ 60 °C; 5 min @ 95 °C; and 40 cycles of 15 sec @ 95 °C followed by 60 sec @ 60 °C. The human acidic ribosomal protein (hARP) gene, a housekeeping gene, will be used to normalize each sample and each gene. The data will be analyzed and samples quantitated by the software provided with the Applied Biosystems PRISM 7700 (Sequence Detection Systems, ver.1.7).

1. **GENE EXPRESSION**

Gene chip studies will be performed as previously published by our group (Ostreicher et al, *The Pharacogenomics Journal,* 2001, 1, 272-287). Total RNA will be isolated from one half of a 6 mm full thickness skin biopsy using the Rneasy mini kit (Qiagen) and amplified using RiboAmp OA^TM^ RNA Amplification Kit (Arcturus). Labeled microarray targets will be prepared, and these will be hybridized to oligonucleotide arrays comprised of 12 000 known human genes (HuGeneU95 Av2, Affymetrix), for detection of fluorescence. Data analysis will be performed on raw fluorescent intensity values using GENECHIP 3.2 software (Affymetrix) or GeneTraffic^TM^.

1. **SATURATION ASSAY**

This competitive assay is performed by incubating PBMCs obtained during and after treatment with an anti-CD11a antibody (clone 25.3, Immunotech) which recognizes a different epitope than efalizumab. Stained cells will be analyzed by FACS to determine the percent binding with efalizumab.

1. **VLA-4 BINDING TO FIBRONECTIN AND ICAM-1**

Our laboratory will collaborate with Dr. Tom Kupper to confirm a previous finding in a small group of patients of decreased binding of T-cells to fibronectin, a VLA-4 ligand.

1. **T-CELL ACTIVATION ASSAYS**

T-cell activation will be assessed using a variety of techniques that are described below. We will need to work out methods so that down-regulation of surface receptors , e.g., LFA-1, TCR chains, and CD2, is maintained during an in vitro assay. Short-term activation-related assays (activation assessed within 4-6 hours) can probably be done on purified cell populations, but longer term assays will probably need to be done in whole blood preps or in purified cell populations to which autologous serum or efalizumab antibodies at an expected serum concentration is added. In turn, we need to establish a time-course for re-expression of various surface proteins after incubation in vitro with cells “washed” of exogenous or free efalizumab. Since we have not been able to reproduce receptor down-regulation by in vitro incubation of PBMC preps with exogenous efalizumab, we must work out re-expression kinetics and assay conditions on the first several patients that will be entered into this trial.

# CD3/CD28 DYNABEADS

: T-cells will be stimulated using CD3/CD28 Dynabeads, which are uniform, superparamagnetic, polystyrene beads coated with a highly optimized mixture of CD3 and CD28 antibodies. The concurrent presentation of both antibodies to one T-cell results in T-cell activation and expansion. To assess activation, we will stain cells for activation-related markers such as CD69 (shown below) or we will assess activation by measuring cycles of divisions as follows

T-cells will be stained with carboxyfluorescein diacetate succinimidyl ester (CFSE), a fluorescein-related dye that passively diffuses into cells and reacts with intracellular amines. During division, the dye-protein complexes are divided equally between daughter cells, permitting eight to ten discrete generations to be identified.

The figure above shows T-cell activation, as measured by CD69 expression, in

control and Dynabeads treated cells.

This figure above shows lymphocytes cultured without activation and stained with CFSE as compared to cells activated with the Dynabeads and stained with CFSE. The majority of lymphocytes have undergone three rounds of division based on CFSE.

# CD2 ANTIBODIES (T11.1 & T11.2)

# Since CD2 is down-regulated by efalizumab, we want to determine how this transmodulation affects T-cell activation.

# STAPHYLOCOCCAL ENTEROTOXIN D (SED)

We will explore the possibility of conducting experiments using SED to activate T-cells. According to previously published material (Chan, 1991), Raji cells are coated with staphylococcal enterotoxin D at 100ng/ml then added to an equal number of Jurkat cells. The cells are rapidly sedimented to initiate cell contact and SED stimulation. The Raji B cells were shown to present SED remarkably well to the Jurkat cells, leading to cell activation. If T-cell activation by this superantigen (in conjunction with Raji cells) is affected by efalizumab administration, we may have a workable system with which it can be determined whether formation of the immune synapse is affected by LFA-1 down-regulation.

# CALCIUM FLUX EXPERIMENT

A change in calcium level is a well-defined component of signal transduction, at the onset of T-cell activation. Fluo-3 AM shows a large fluorescence intensity increase in response to calcium binding and has been extensively utilized for flow cytometry calcium flux imaging. Peripheral blood mononuclear cells will be loaded with Fluo-3 AM. These will then be acquired through a flow cytometer, for baseline reading. The cells will then be activated with CD3 antibodies and a combination of CD2 antibodies (T11.1 and T11.2). An increase in Fluo-3 AM fluorescence after activation would demonstrate calcium flux.

The figures above are from previous work done in our laboratory that shows a decrease in calcium flux with the addition of efalizumab in vitro.

1. **FLOW CYTOMETRY**

Flow cytometry analysis will be performed on peripheral blood mononuclear cells to measure expression of adhesion and activation-related proteins, such as CD3, CD11a, CD18, VLA-4, TCRα/β, CD8, CD4, CD2, CD25, TNF-α and CD69. These will be studied during treatment and follow-up phase.

Cytokine production of circulating T-cells will also be measured by flow cytometry. The following cytokines will be analyzed: γ-IFN, IL-4, IL-10, TNF-α, and IL-2.

Flow cytometry will be performed on whole blood to characterize the expression of cell surface markers on circulating leukcoytes eg.

B cells CD19 or CD20

NK CD2, CD16, CD56, (CD3-)

NK-T cells CD3, CD94, CD161, CD1d ligand tetramer

Memory T-cells CD3, CD45RA, CD45RO, CCR7, CD27, CD28

Neutrophils CD11b and CD14 (CD3-)

Monocytes CD2, CD14, CD11c/CD18 (CR4), CD58

Dendritic cells CD83, CD86, CD123

Integrins CD49d/CD29 (VLA-4), CD11a/CD18 (LFA-1),

CD11b/CD18 (Mac-1, CR3)

Changes in specific populations of antigen-specific (CMV-positive) T cells may be examined by tetramer staining.

Other markers may be studied as they become available.

We also plan to measure LFA-1 in different conformational states on circulating leukocytes.

1. **APOPTOSIS**

Apoptosis in circulating cells will be measured by annexin+ PI- staining by flow cytometry. Apoptosis at the tissue level will be measured by the TUNEL reaction.

1. DENDRITIC CELL STUDIES

9A: To characterize the phenotype and genomics of peripheral blood DCs in patients with psoriasis and those receiving efalizumab (anti-CD11a) therapy.

1. *Define subsets of DCs and activation states in peripheral blood by FACS*

The classification of DCs in peripheral blood and tissues is evolving. We plan to use a panel of antibodies to enumerate DCs and their activation states in peripheral blood in patients with psoriasis (pre-treatment) and compare with those receiving efalizumab at an intermediate timepoint. The FACS panel to study peripheral leukaoytes includes markers such as: HLA-DR, Lin cocktail (CD3, CD15, CD56, CD19, CD20, CD14), Cd1a/b/c, CD2, CD14. CD16, CD25, CD33, CD34+, CD11a/b/c, CD52, CD68+, CD83, CD86, CD-205 (DEC-205), CD-206 (MMR), CD-207 (langerin/lag), CD-208 (DC-LAMP), CD-209 (DC-SIGN), CD123, BDCA-2, BDCA-3, BDCA-4, and CD119, Factor XIIIa. Previously discussed methods of DC classification will be compared. Our aim is to find a specific marker or set of markers that might identify these cells.

1. *Perform genomic studies (± real time RT-PCR) of DCs from peripheral blood*

We will FACS-sort DCs from the peripheral blood using the classic HLA-DR+ Lin- definition. However, as we asses the above panel to clarify DC subpoulations, other definitions may be more useful to FACS sort and analyze, such as HLA-DR+ CD14- CD33+. This will be performed on psoriatics (pre-treatment) and compare with those on efalizumab at an intermediate timepoint (week 4 or 8). 50mls heparinized whole blood will yield 50 000 cells (1% of 50 x 10^6^ PBMC), which should yield sufficient RNA for amplification. PBMCs will be frozen in RNA lysis buffer, and stored at –80°C until required for analysis.

Based on recent studies, there is a long list of interesting inflammatory genes to examine by gene chip and confirm by real time RT-PCR, as outlined in the table below

| **Cytokines** | **Chemokines** | **DC markers/products** | **Other** |
| --- | --- | --- | --- |
| IL-1β | CCL 2 (MCP-1) | Factor XIIIa | lipase A |
| IL-6 | CCL 3 (MIP-1α) | CD11a/b/c | lysosomal acid |
| IL-8 | CCL 4 (MIP-1β) | CD33 | ICAM-1 |
| IL-10 | CCL 5 (RANTES) | CD 205 (DEC-205) | NFκB |
| IL-12 p23 | CCL 7 (MCP-3) | CD 206 (Mannose R) | Rel-B? |
| IL-12(23) p40 | CCL17 (TARC) | CD 207 (langerin) |  |
| IL-23 p19 | CCL18 (PARC) | CD 208 (DC-LAMP) |  |
| TNF-α | CCL19 (MIP-3β) | CD 123 (IL-3Rα chain) |  |
| IFN-γ | CCL20 (MIP-3α) |  |  |
|  | CCL 21 (6Ckine) |  |  |
|  | CXCL 9 (MIG) |  |  |
|  | CXCL 10 (IP-10) |  |  |
|  | CXCL 11 (I-TAC) |  |  |

Total RNA will be isolated from 50,000 cells using the Rneasy mini kit (Qiagen) and amplified using RiboAmp OA^TM^ RNA Amplification Kit (Arcturus). Labeled microarray targets will be prepared, and these will be hybridized to oligonucleotide arrays comprised of 12 000 known human genes (HuGeneU95 Av2, Affymetrix), for detection of fluorescence. Data analysis will be performed on raw fluorescent intensity values using GENECHIP 3.2 software (Affymetrix) or GeneTraffic^TM^.

For confirmation RT-PCR studies, we already have many of the primer-probe sets to proceed, and will design or obtain additional published sets as required.

# 9B. To characterize lesional DCs *in vivo* in patients with psoriasis and those receiving efalizumab (anti-CD11a).

1. *Characterize inflammatory mediator production by tissue DCs in patients on efalizumab therapy*

Pre-treatment and treatment skin biopsies of patients on the efalizumab trial will be double stained with fluorescence labeled CD11c and inflammatory mediators such as TNF-α, iNOS, IL-23p19, IL-23p40. We are able to add selected fluorescence labels to primary antibodies by the Alexa Kit (Molecular Probes), and visualize double staining with fluorescence microscopy. We plan to refine this by using the confocal microscope for future experiments. Biopsies are obtained at weeks 0, 2, 6 and 12.

1. *Define subsets of lesional DCs and activation states in cell suspensions by FACS in patients on efalizumab therapy*

The panel of antibodies outlined under Aim 1 will be used to enumerate DCs and their activation states *in situ* in patients with psoriasis (pre-treatment) and compare with those receiving efalizumab. Shave biopsies are obtained at weeks 0 and 2 for generating cell suspensions for this FACS analysis. Cell suspensions are obtained after a 4 hour culture using a method of Fczerenzi et al developed in our laboratory (REF). This yields approximately 10^5^ cells. Half of these are used for FACS and remainder frozen.

1. *Perform genomic studies of lesional tissue in patients on efalizumab therapy*

Lesional RNA will be generated from patients on therapy, gene-expression will be studied on gene chips, and confirmed by real-time RT-PCR where appropriate. Biopsies are obtained at weeks 0, 2, 6 and 12. Total RNA will be isolated from one half of a 6 mm full thickness skin biopsy using the Rneasy mini kit (Qiagen) and amplified using RiboAmp OA^TM^ RNA Amplification Kit (Arcturus). Gene array studies are outlined above.

The viability of laser micro-dissection to enable detailed analysis of CD11c+ cells will be pursued. Mediator production on individual cells will be assessed, and if sufficient numbers are obtained, we will perform genomic studies.

9C. To characterize how eflalizumab affects the interaction between T cells and DCs in psoriasis *in vitro*.

For these experiments, buffy coats of normal volunteers will be purchased from NY Hospital Red Cross Blood Bank. T cells will be removed and saved and monocyte-derived DCs will be generated.

1. *Determine whether anti-CD11a ligation in T cells blocks T cell-directed activation and/or differentiation of DCs*

Efalizumab will be bound to T cells, and co-cultured with immature DCs. Their ability to mature DCs will be studied by FACS and modified MLR (CSFE addition). This experiment will be repeated with cells transfected with CD40L (ATCC G28-5), and with TNF blockers.

1. *Effect of addition of efalizumab on DC phenotype, function, apoptosis, and inflammatory gene expression in vitro.*

Efalizumab will be added to immature and mature DCs directly to see the effect on phenotype (FACS), function (modified MLR), apoptosis (Annexin/ PI FACS staining), and gene expression (Gene array ± RT-PCR).

1. *Determine the role of DCs in the down-regulation of components of the immune synapse.*

Fluorescent labeled efalizumab (label using the Alexa kit) will be bound to T cells. These T cells will be cultured with immature and mature DCs to observe whether these complexes are ingested by fluorescent microscopy.

Pitfalls: In vitro affects of efalizumab may not be the same as in vivo. Thus applicability needs to be considered when drawing conclusions from these in vitro experiments. They may need to be repeated ex-vivo if possible.

# Appendix G

## Patient Information (taken directly from its label)

**RAPTIVA (Rap-TEE-vah) (efalizumab)**

**for injection, subcutaneous**

Read the Patient information that comes with RAPTIVA™ (efalizumab) before you start using it and each time you get a refill. There may be new information. This information does not take the place of talking with your healthcare provider about your medical condition or treatment. It is important to remain under a healthcare provider’s care while using RAPTIVA. **Do not change or stop treatment without first talking with your healthcare provider.** Talk to your healthcare provider or pharmacist if you have any questions about RAPTIVA.

WHAT IS THE MOST IMPORTANT INFORMATION I SHOULD KNOW ABOUT RAPTIVA?

**RAPTIVA can decrease the activity of your immune system.** Therefore, people using RAPTIVA may have an increased chance of getting:

- **Serious infections**. Some infections could become serious. If you have an infection, tell your healthcare provider before you start using RAPTIVA. If you get an infection that does not go away while taking RAPTIVA, tell your healthcare provider right away.
- **Cancers**. Many drugs that decrease the activity of the immune system can increase the risk of cancer. If you have had cancer you should tell your healthcare provider before you start taking RAPTIVA. The role of RAPTIVA in the development of cancer is not known.
- **Low platelet counts (thrombocytopenia)**. Platelets help your blood clot. Low platelets give you a higher chance for bleeding. Call your doctor right away if you have increased bruising or bleeding. Your healthcare provider may do regular blood tests to check your platelets while you are taking RAPTIVA.
- **Worsening of psoriasis**. Some patients have had severe worsening or new forms of psoriasis while taking RAPTIVA or after stopping RAPTIVA. Tell your healthcare provider right away if your psoriasis gets worse or if you see any new rashes during or after treatment with RAPTIVA.

**You should not receive vaccines while using RAPTIVA.** RAPTIVA may prevent a vaccine from working. Talk to your healthcare provider if you need to receive a vaccine while using RAPTIVA.

**WHAT IS RAPTIVA?**

RAPTIVA is a medicine usd to treat adult patients with moderate to severe plaque psoriasis who can be treated with medicines that affect the whole body (systemic therapy) or with phototherapy.

RAPTIVA is a man-made protein that is like proteins made in the body called antibodies. Antibodies fight disease in the human body. RAPTIVA may decrease the skin changes in the body that are the main problems of moderate to severe plaque psoriasis.

RAPTIVA has not been studied in children under 18 years of age.

**WHO SHOULD NOT USE RAPTIVA?**

Do not use RAPTIVA if you have ever had an allergic reaction to RAPTIVA.

**Before using RAPTIVA, tell your healthcare provider**

1. **about the following medical conditions:**

- **If you are pregnant, planning to become pregnant, or become pregnant while using RAPTIVA.** It is not known if RAPTIVA can harm your unborn baby. If you become pregnant while taking RAPTIVA, notify your healthcare provider immediately. You and your healthcare provider will have to decide if RAPTIVA is right for you during pregnancy. If you use RAPTIVA when you are pregnant, ask your healthcare provider how you can be on the RAPTIVA pregnancy registry.
- **If you are breast feeding.** It is not known if RAPTIVA passes into your milk. It may harm your baby. You will need to decide whether to use RAPTIVA or breast feed, but you may not do both.
- **If you have any infections** (see **WHAT IS THE MOST IMPORTANT INFORMATION I SHOULD KNOW ABOUT RAPTIVA?**).
- **If you have immune system problems;**

1. **about all the medicines you take including prescription and nonprescription medicines, vitamins, and herbal supplements.** It is not known if RAPTIVA and other medicines affect each other. **Especially, tell your healthcare provider if you are using:**

- **Other medicines or treatments for your psoriasis.**
- **Medicines called immunosuppressives or any medicine that affects your immune system.** Ask your healthcare provider or pharmacist if you are not sure if any of your medicines are immunosuppressives.

### HOW SHOULD I USE RAPTIVA?

- Ask your healthcare provider or pharmacist if you have any questions about using RAPTIVA.
- Use RAPTIVA exactly as prescribed by your healthcare provider. Your dose of RAPTIVA is based on your body weight. Tell your healthcare provider if your weight changes. Do not change your dose without talking to your healthcare provider. Do not stop using RAPTIVA without talking to your healthcare provider.
- RAPTIVA is injected under the skin (subcutaneous) on your upper leg (thigh), upper arm, abdomen, or buttocks once a week. Change (rotate) your skin injection site with each injection.
- Use RAPTIVA the same day each week. If you miss your dose of RAPTIVA contact your healthcare provider right away.
- See your healthcare provider regularly while using RAPTiVA. Do not miss your appointments. Your healthcare provider may do blood tests including platelet counts before and during treatment with RAPTIVA to check its affects on your body.

**WHAT SHOULD I AVOID WHILE USING RAPTIVA?**

**Unless directed by your healthcare provider, do not:**

- take other medicines called immunosuppressives.
- take treatments called phototherapy.

**You should not receive vaccines while using RAPTIVA**. Talk to your healthcare provider if you need to receive a vaccine while taking RAPTIVA (see **WHAT IS THE MOST IMPORTANT INFORMATION I SHOULD KNOW ABOUT RAPTIVA**).

**WHAT ARE THE POSSIBLE SIDE EFFECTS OF RAPTIVA?**

**RAPTIVA can cause serious side effects including the following** (see **WHAT IS THE MOST IMPORTANT INFORMATION I SHOULD KNOW ABOUT RAPTIVA**):

RAPTIVA can affect your immune system and might cause:

- **Serious infections**
- **Cancers**
- **Low platelet counts (thrombocytopenia)**
- **Worsening of psoriasis**

**The most common side effects of RAPTIVA** include headache, chills, fever, nausea, and muscle aches. These reactions usually happen within the first 48 hours following RAPTIVA injection, and often decrease after the first few weeks of use of RAPTIVA. Back pain, joint pain, and swelling of the arms or legs (peripheral edema) can also happen with RAPTIVA. Talk to your healthcare provider about any symptoms that bother you.

If you get any side effect that concerns you or if you get an infection, call your healthcare provider.

These are not all the side effects of RAPTIVA. For more information, ask your healthcare provider or pharmacist.

### GENERAL INFORMATION ABOUT RAPTIVA

Medicines are sometimes prescribed for conditions that are not mentioned in patient information leaflets. Do not use RAPTIVA for a condition for which it was not prescribed. Do not give RAPTIVA to other people, even if they have the same symptoms you have. It may harm them.

This document summarizes the most important information about RAPTIVA. If you would like more information, talk with your healthcare provider. You can ask your healthcare provider or pharmacist for information about RAPTIVA that is written for health professionals. For more information, you can also call 1-877-RAPTIVA (toll free).
